# Dual-function silver nanoparticles catalyst supported on ZnO nanoparticles from plant extract: high-TOF hydrogen generation and fast photocatalytic tetracycline removal

**DOI:** 10.1039/d5ra09874b

**Published:** 2026-04-01

**Authors:** Sayyar Ali Shah, Shah Faisal Mohammad, Abida Batool, Muhammad Saad Riaz, Azhar Abbas, Shoaib Akhtar, Ibrahim A. Shaaban, Umar Nishan, Hanbing Song

**Affiliations:** a School of Medical Sciences, Shandong Xiehe University Jinan Shandong Province P.R.China; b Department of Chemistry, Superior University Lahore Lahore 54000 Pakistan; c Institute of Chemistry, University of Sargodha Sargodha 40100 Pakistan shoaibbalkachem42@gmail.com; d Government Ambala Muslim Graduate College Sargodha 40100 Pakistan azhar.ramzan@uos.edu.pk; e Department of Chemistry, Faculty of Science, Research Center for Advanced Materials Science (RCAMS), King Khalid University P.O. Box 960 Abha 61421 Saudi Arabia; f Department of Chemistry, Kohat University of Science and Technology Kohat 26000 KP Pakistan

## Abstract

A green hybrid AgNPs@ZnO nanocomposite (NC) was prepared, employing *Cyperus scariosus* root extract as a natural reducing and stabilizing agent. The prepared nanostructure was thoroughly investigated *via* UV-vis, Fourier Transform Infrared Spectroscopy (FTIR), X-ray Diffraction (XRD), Scanning Electron Microscope (SEM), Energy Dispersive X-ray Spectroscopy (EDS), Thermogravimetric Analysis (TGA), and Brunauer–Emmett–Teller (BET) analyses and confirmed the success of Ag deposition and the mesoporous support with considerably increased surface area and stable nature. The dual-functional catalytic activity of AgNPs@ZnO was also tested for hydrogen evolution reaction (HER) at low temperature with Formic Acid (FA) as a hydrogen source and the photo-catalytic degradation of Tetracycline (TC) under solar light. The catalytic dehydrogenation of formic acid (FA) on Ag@ZnO NCs was systematically investigated under various experimental conditions. The catalyst exhibited outstanding efficiency at pH 4 with a TOF of 3935 h^−1^ owing to abundant protons and a thermodynamically favorable Ag–ZnO interfacial synergy. The FA/Sodium Format (SF) molar ratio (3 : 1) and the catalyst amount (15 mg) were optimized, whereas water was evaluated as being more effective than DMF or methanol. These results reveal that Ag@ZnO NCs are highly active catalysts for the efficient, selective, and recyclable hydrogen generation from FA under an optimal reaction condition. Photocatalytic degradation of TC was carried out using AgNPs@ZnO, showing an efficient and fast removal (∼99% over 45 min) following pseudo-first-order kinetics, with a low Activation Energy (*E*_a_) value (4.94 kJ mol^−1^) and favorable thermodynamic parameters (Δ*H* = 2.32 kJ mol^−1^ and Δ*G* ≥ 27 kJ mol^−1^). The studies of scavenger and band-edge showed that superoxide (˙O_2_^−^) and hydroxyl (˙OH) radicals were major Reactive oxygen species (ROS) in the system, and were enhanced by the synergistic Ag–ZnO effects. The good recyclability of the nano catalyst revealed the potential of NCs for practical applications toward energy and the environment.

## Abstract 2

Green-synthesized AgNPs@ZnO using *Cyperus scariosus* extract shows dual functionality with efficient H_2_ generation (TOF 3935 h^−1^) from formic acid and ∼99% tetracycline degradation under solar light, driven by strong Ag–ZnO synergistic effects.

## Graphical abstract

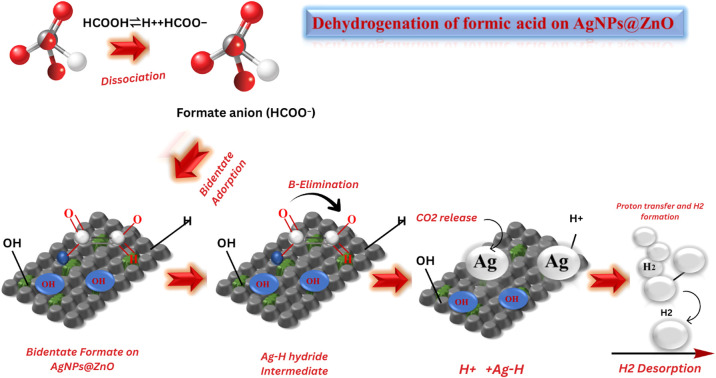

## 1. Introduction

Hydrogen (H_2_) is one of Earth's most abundant energy carriers and has been established as one of the most favorable, clean, and renewable energy sources in the search for a durable, sustainable alternative (with an energy density of 142 MJ kg^−1^), mainly due to both its high energy content and environmentally friendly byproduct (water).^1^ Hydrogen is a zero-carbon fuel and can fuel cells emission-free and in combustion plants without releasing greenhouse gases, and is directly applicable to the transport, power, and chemical sectors.^2,3^ The exploration of high-performance, non-expensive Hydrogen Evolution Reaction (HER) catalysts that are capable of performing well at room temperature is still a key milestone for large-scale hydrogen production and its incorporation into sustainable energy systems.^4^

Hydrogen can be generated by physical and chemical methods. Physical approaches, including water electrolysis, thermolysis, and photo-catalysis, generally require high energy consumption or sophisticated reactor types, which are, however, less economically and technically feasible.^5,6^ In comparison, chemical hydrogen carriers, such as FA (HCOOH), ammonia (NH_3_), sodium borohydride (NaBH_4_), and boranes, provide on-demand, tunable release of hydrogen at marginal temperatures, which can be accommodated by current infrastructure.^7–9^ Of these, FA (Formic Acid) has attracted the most attention owing to its having an extremely high hydrogen content (4.4 wt%), liquid-phase stability, and convenient handling, as well as being able to produce hydrogen by selective catalytic dehydrogenation (HCOOH → H_2_ + CO_2_).^10–12^ Such an optimal catalytic system is considered an attractive source of hydrogen because of its high cost-effectiveness for low-temperature HER.

Nano Particles (NP) catalysts are undergoing a dramatic development in the area of energy, together with environmental catalysis.^13^ With their high surface-to-volume ratio and the possibility to tune their electronic properties, Nano Particles (NPs) can possess increased catalytic activity, stability, and surface reactivity.^14^ Of these, noble metal NPs like silver (Ag) NPs, in particular, have been widely investigated on account of the surface plasmon resonance, electron-trapping ability, and catalytic activity.^15,16^ But they can still be enhanced further by depositing them over the semiconductor metal oxides, such as ZnO.^17^

ZnO, a large bandgap (3.3 eV) semiconductor, has found applications in photo-catalysis, sensors, and antimicrobial activity for its multi-functionality.^18,19^ The low price, ready availability, and good chemical stability of C have generated interest as a support for a catalyst.^20^ AgNPs@ZnO Nano Composites (NCs) show enhanced catalytic activity through Ag NPs decoration with a higher electron transfer rate, more active surface sites and decreased electron hole recombination.^21,22^ These composites not only improve the catalytic activity and stability, but also reduce the *E*_a_ for hydrogen evolution in mild conditions, which is very important for practical HER applications.^23^

In addition, apart from the sustainable synthesis and intrinsic dual nature, the AgNPs@ZnO Nano composites (NC) possess a plethora of other plausible mechanistic and practical merits that accentuate its applicability toward energy and environmental arenas. The Schottky junction between Ag and ZnO can promote effective charge separation and restrain the electron–hole recombination, thus benefitting the enhancement of photo-catalytic and electro-catalytic performance.^24^ Under photo-catalysis, they found that hydroxyl and superoxide radicals are the key oxidants responsible for Tetracycline (TC) degradation, while Ag NPs display to be the catalytic sites for hydrogen evolution through FA dehydrogenation.^25^ Also, the NC shows good structural stability and reusability under reaction conditions, and is therefore, a good candidate for the sustainable long-term use.^26^ The inclusion of plant-based green synthesis eliminates the use of hazardous chemicals and reduces the overall cost of the synthesis process with emphasis on the environmental and economic appropriateness of AgNPs@ZnO for simultaneous pollutant decomposition and clean hydrogen production.^27^

Nanomaterials are also being investigated to efficiently remediate water pollution from pharmaceutical pollutants. TC, a broad-spectrum antibiotic that has been used extensively in both human and veterinary communities, is commonly observed in the surface water, wastewater, and soil owing to its incomplete metabolism and inappropriate disposal.^28^ Due to its long-term existence in water, it brings serious ecological and health risks, such as antimicrobial resistance and aquatic organism toxicity. Because of its aromatic and functionalized structure (hydroxyl, amide, and dimethyl amino groups), TC is difficult to remove *via* conventional wastewater treatment technologies, and the development of more advanced degradation technologies is required.^29^

Many methods have been explored for the degradation of TC, such as adsorption, photo-catalysis, Advanced Oxidation Processes (AOPs), and nanomaterial-mediated degradation.^30^ Of those technologies, NP-based photo-catalysis has been gaining a lot of attention and interest in recent times due to its high efficiency, reusability, and possibility of operating under ambient or visible-light conditions.^31^ Therefore, the designed AgNPs@ZnO can act as a dual-role nanomaterial for the selective degradation of TC photo-catalysis and hydrogen production under similar reaction conditions, achieving simultaneous treatment of energy and environmental problems.^32^

Synthesis and applications of AgNPs@ZnO NC were reported in many studies. For instance, Fahmy *et al.* reported that AgNPs-coated ZnO had bacteriostatic properties that prevent the formation of high levels of ROS.^33^ Kheirabadi *et al.* (2019)^34^ and Mohammadzadeh *et al.* (2015)^35^ showed that the Ag–ZnO systems have been used for better photo-catalytic degradation of organic dyes for the faster electron–hole separation and better light absorption.^15^ Nevertheless, these methods involve conventional chemical synthesis methods and are usually accompanied by the use of toxic solvents and high energy consumption. Green synthesis methods have gained momentum lately, responding to increasing environmental awareness.^36^ Osuntokun *et al.* (2019) were prepared under the influence of aqueous Brassica oleracea extract,^37^ on the other hand, Khane *et al.* (2022) also used Citrus limon for the synthesis of AgNPs.^38^ Iqbal *et al.* Azadirachta indica extract for green synthesis of Ag and ZnO material with significant antimicrobial activity.^39^ Ahmad *et al.* evidenced a catalyst for room-temperature sustainable hydrogen generation from FA.^40^

Although plant-borne Ag–ZnO NCs have demonstrated high-quality TC degradation and Ag-based catalysts have efficiently participated in FA dehydrogenation, no evidence has been provided that constructs an individual NC that performs both duties under green fabrication.^41^ Most of the reported systems are used for one function application and require expensive noble metals (Pt, Pd) or an environmentally unfriendly process.^42^ This demonstrates a large development space for a dual-functional and sustainable catalyst which can be applied in the low-temperature HER reaction in FA as well as the photo-catalytic decomposition of pharmaceutical contaminants (such as TC) under natural light irradiation.^43^

Although of Ag/ZnO photocatalysts have been investigated, most are fabricated through chemical methods and can serve for either pollutant degradation or H-2 production. In the present study, we report on a green preparation of AgNPs@ZnO nanocomposite using *C. scariosus* extract and exhibit its excellent bifunctional performance for formic acid dehydrogenation and tetracycline degradation under solar irradiation.

In the current work, we fill this research gap by fabricating an eco-friendly synthesized AgNPs@ZnO NC with a plant extract acting as the natural reducing and stabilizing agent. The present extract-induced synthesis process offers a green synthesis protocol without involving hazardous chemicals towards the induction of well-dispersed Ag NPs on the ZnO surface. The synthesized NC was well characterized and employed for dual catalytic activities: (i) electrocatalytic HER at low temperature utilizing FA as a model H source, and (ii) photo-catalytic degradation of TC under UV-light irradiation. The introduction of AgNPs is supposed to improve the charge separation and surface reactivity, and ZnO is an excellent, stable photo-catalytic support matrix. The report presents a sustainable and multifunctional catalyst for the simultaneous handling of clean energy production and environmental pollutant remediation, and pushing forward the development of integrated green technologies.

## 2. Materials and methods

### 2.1 Materials and chemicals

The reagents and solvents were of analytical grade and used without any purification. Zinc acetate dihydrate [Zn(CH_3_COO)_2_·2H_2_O], silver nitrate (AgNO_3_), sodium borohydride (NaBH_4_), and *N*, *N*-dimethylformamide (DMF) were obtained from Sigma-Aldrich (USA). The ethanol and organic solvents were purchased from Lab Scan (Thailand). The synthesis and photo-catalytic experiments involved all reagents and deionized distilled water. *Cyperus scariosus* was obtained in the local area, and the roots of *Cyperus scariosus* were used as the source of green in the production of ZnO NPs. TC hydrochloride was purchased from Sigma-Aldrich, and it was utilized as a model pollutant in the degradation experiments.

### 2.2 Preparation of plant extract

The fresh root of *Cyperus scariosus* was moistened under a lot of distilled water to remove the soil and dust, and dried in shade over a period of 7–10 days. The dried roots were powdered, and 20 g of the powder was mixed with 200 mL of deionized water. The suspension was then heated to 80 °C, after which the filtration through Whatman No. 1 filter paper was done in 30 min. The extracted plant extract was stored at the refrigerator temperature (4 °C) and applied within 48 h as a green reducing and capping agent in the preparation of ZnO NPs.

The plant extract acted as a natural reducing and stabilizing agent during the synthesis. The extract phytochemicals (polyphenols and flavonoids) mediated the Zn^2+^ ions complexation, controlled nucleation rate, and capped the surface of growing ZnO nanoparticles through surface adsorption. This bio-derived method averted particle agglomeration and facilitated the fabrication of homogeneous stable ZnO nanostructures, in the absence of synthetic capping agents.

### 2.3 Synthesis of the catalyst

#### 2.3.1. Synthesis of ZnO NPs

A typical method of green synthesis of ZnO NPs under the influence of *Cyprus scariosus* plant extract. 2.5 g of the zinc acetate dihydrate dissolved in 100 mL of deionized (DI) water, which was then dropped slowly under constant magnetic stirring at room temperature with 25 mL of the plant extract suspension. The process was stirred until the reaction mixture reached 70 °C. After this time, an additional 30 min of stirring was carried out. A brown precipitate was observed, and this is typical of the ZnO precursors. The product was centrifuged, washed with plenty of distilled water, and dried at 80 °C. To obtain crystalline ZnO NPs, the dried powder was burned in a muffle furnace at 450 °C over a period of two hours and ground and stored under the case of the study.

#### 2.3.2. Preparation of Ag NPs decorated ZnO (Ag NPs@ZnO)

The reduction of ZnO 5wt% Ag-loading anchored Ag NPs to its surface. In brief, 0.5 g of ZnO NPs was added to 100 mL of DI water through ultrasonication for 15 minutes in order to produce an even suspension. This was followed by a slow addition of 0.01 M AgNO_3_ solution to the ZnO suspension, which was stirred. Ag^+^ ions were adsorbed at the ZnO surface, and the slow addition of 0.01 M AgNO_3_ was put into the solution. The reducing agent of a 0.01 M freshly prepared NaBH_4_ solution was added slowly to the stirring solution, and the mixture was left to react for the next 30 min. Ag^+^ was formed as evidenced by the change in the color of the solution. The reaction was also extended to an extra hour. The product was then centrifuged at a speed of 5000 rpm for 15 min and washed with ethanol and distilled water a few times, followed by drying the product in a 60 °C oven overnight to acquire the Ag NPs@ZnO NCs. The powdered catalyst obtained was put in a desiccator to characterize and do photo catalytic analysis.

### 2.4 Characterization techniques

Through the various methods of operation, structural, morphological, thermal, optical, and surface studies, a general account of the Ag NPs@ZnO NC was established by use of various tools of analysis. The optical properties were achieved by carrying out UV-vis spectroscopy (Shimadzu UV-2600, Japan) in the wavelength range of 200–800 nm, and Tauc plots were applied to calculate the band gap energy. A PANalytical X'Pert PRO diffractometer (PANalytical, Netherlands) with a Cu Ka X-ray (*l* = 1.5406 A) in the 2 theta range (10°–80°) was used to do the Powder X-ray Diffraction (XRD). Fourier Transform Infrared Spectroscopy (FTIR) was used to characterize the functional group of the nano-FBPs in the wavenumber range, 4000–400 cm^−1^ (Bruker Alpha II, Germany).

Morphologies and size of surface particles were examined using Scanning Electron Microscope (SEM), using JEOL JSM-6490LA microscope (Japan), and elemental composition and Ag dispersion were checked using Energy Dispersive X-ray Spectroscopy (EDS) with the SEM. To determine the thermal stability of the developed samples, Thermogravimetric Analysis (TGA) was performed on a PerkinElmer Thermo Gravimetric Analysis 4000 (USA) under nitrogen atmosphere with a flow rate of 20 mL min^−1^ at a temperature ranging between 30 and 800 °C at a heating rate of 10 °C min^−1^. The pore size distribution, pore volume, and the specific surface areas were calculated through the nitrogen adsorption–desorption isotherms utilizing the Brunauer–Emmett–Teller (BET) method, which was done on a Micrometrics ASAP 2020 analyzer (USA). Degassing of samples was done at 150 °C in advance of the analysis.

### 2.5 Photo-catalytic degradation of TC

Ag NPs@ZnO was also tested in its photo-catalytic activity on the degradation of TC when exposed to natural sunlight. A 20 mg L^−1^ solution of TC (250 mL) was made, and 0.1 g of the catalyst was added to it. The reaction mix was stirred and shaken in the dark over 30 min to bring the system to adsorption–desorption equilibrium and then was subjected to the sun in the UV-vis spectrophotometer at a wavelengths of 360 nm. 5 mL of the suspension mixture was sampled at various intervals (0, 5, 10, 20, 25, 30, 35, 40 and 45 min) and the catalyst particles were separated and the supernatant solution measured at the degree of degradation (*η*) was calculated by equation:1
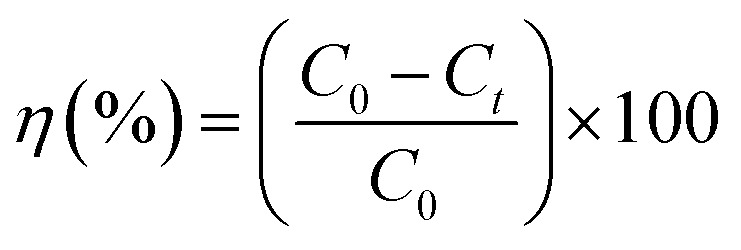
*C*_0_ = initial concentration of TC and *C*_*t*_ = concentration of TC *versus* time, respectively. The effect of PH (2–10) and temperature (318–368 K) was examined. The pseudo-first-order equation was used to describe the kinetics of the reactions. Gibbs free-energy (Δ*G*) and entropy (Δ*S*), as well as thermodynamic parameters (*E*_a_, enthalpy (Δ*H*)), have been estimated. The active species of the degradation system confirmed by scavenger experiments were methanol (OH˙ scavenger) and superoxide dismutases (˙O_2_^−^ scavenger).

#### 2.5.1. Catalytic dosage optimization

Experiments with varied quantities of Ag NPs@ZnO catalyst were also conducted to determine a good dose of catalyst that would give an optimal degree of degradation efficiency. The quantity of catalyst and 250 mL of 20 mg L^−1^ of TC solution were then added to each system independently at the same solar irradiation. All other parameters were homogeneous in terms of pH, temperature, and time of exposure. Spectroscopic monitoring of the reactions in terms of UV-vis was carried out, with the degradation yields calculated. An optimal dose of nano size was obtained as the dose that gave maximum degradation with minimum expenditure of the catalyst, and which offered a more cost-effective and efficient approach.

#### 2.5.2. Silver loading optimization

Photo-catalytic activity depends on the amount of the Ag NPs loaded on the ZnO surface. To examine this effect, Ag NPs@ZnO NCs were synthesized using various concentrations of silver (1 wt%, 3 wt%, 5 wt%, and 7 wt%). (*Via* conjugation at the particle-surface) The loading was regulated by the change of the concentration of AgNO_3_ used in the synthesis step and reduced/deposited in the same way. The synthesized NCs were then tested on the degradation of TC under the same conditions, and 5 wt% Ag-loaded photo catalyst displayed the highest degradation efficiency and was then used to do all the experiments. This maximum-value extent helped to separate the charge better and inhibit electron–hole recombination in cases where Ag was not over-aggregated.

#### 2.5.3. Effect of solvent medium

To evaluate the influence of the reaction media on the photo-catalytic activity, the different solvents were tested using the same experimental procedure. Solvents that were experimented included are deionized water, ethanol, and *N*, *N*-dimethylformamide (DMF). 0.1 g of Ag NPs@ZnO was dispersed in the total volume of 250 mL of 20 mg L^−1^ TC solution that was collected in the respective solvent in all cases. The degradation process was not disturbed further. The degradation was followed by periodically taking UV-visible absorbance measurements. Deionized water was observed to be the most effective solvent to transfer charge and the least effective to deactivate the photo catalyst, and therefore was used as the control medium of all other experiments.

#### 2.5.4. Effect of initial pH

The pH of the reaction system has a significant impact on the surface charge of the catalyst, pollution ionization, and the formation of ROS. To test the pH effect, 0.1 M NaOH or HCl was added to the initial pH solutions (20 mg L^−1^) to make them acidic (pH 2), basic (pH 6, 8, 10). Ag NPs@ZnO was used in the photo-catalytic degradation process using 0.1 g of Ag NPs@ZnO under the identical conditions of sunlight. The kinetics of the reactions were determined at every pH, with the highest removal efficiency being expressed as the optimal pH. The photo catalyst showed improved photo degradation in weak acidic and neutral conditions due to the adsorption effect of the surface and the large production of HO.

#### 2.5.5. Kinetic and thermodynamic studies

The catalytic reaction model of the degradation process of the photo catalyst was a pseudo-first-order reaction:2
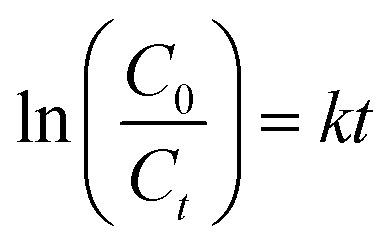
where *k* is the rate constant, *C*_0_ is the initial concentration, and *C*_*t*_ is the concentration at time. It was determined by the slope of the linear plots of ln (*C*_0_/*C*_*t*_) against time. Parameters of thermodynamics, like *E*_a_, enthalpy change (Δ*H*), entropy change (Δ*S*), and Gibbs free energy change (Δ*G*), were the ones obtained through temperature-dependent rate constants. The experiments have been conducted at different temperatures (318 K, 328 K, 338 K, 348 K, and 368 K), and the parameters have been determined by using the Arrhenius and Van't Hoff equations that showed the nature and spontaneity of the degradation reaction.

#### 2.5.6. Turnover frequency (TOF) calculation

The frequency of turnover (TOF) was used to determine the intrinsic catalytic activity of the Ag NPs@ZnO photocatalyst based on the quantity of tetracycline (TC) that was degraded per hour in terms of moles per mole of silver (Ag). TOF was calculated in the following way:3
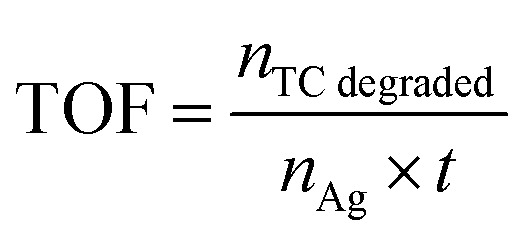
where *n*_TC degraded_ is the moles of TC degraded actually, *n*_Ag_ is the moles of silver in the photo catalyst, and *t* is the time of the reaction in hours. In a normal test, 0.1 g of Ag NPs@ZnO (5 wt% Ag) was used to degrade 250 mL of 20 mg L^−1^ TC solution in the natural sunlight. Data in the introduction of the TOF equation was based on 90% degradation of TC and TC moles degraded, and silver in the reaction mixture was inserted to obtain a normalized activity of the catalyst.

#### 2.5.7. Identification of the reactive species with the scavenger study

Scavenger experiments were conducted to determine the active species that cause photo-catalytic degradation. Methanol (10 mM) was the Hydroxyl radicals scavengers (˙H), and superoxide radicals (˙O_2_^−^) scavengers was superoxide dismutase enzyme. These scavengers were put in the reaction system before the light illumination. The large reduction in the efficiency of the degradation in the presence of scavengers was indicative of the fact that the associated reactive species were the primary active species in the photo-catalytic reaction.

### 2.6 Hydrogen evolution reaction (HER testing)

Evaluation of the HER activity of the samples was performed using the measurement and cell configuration (HER testing). The hydrogen evolution activity upon the Ag NPs@ZnO catalyst was evaluated in a quartz reactor under visible light at low temperature. A 10 vol% FA as a sacrificial proton donor was prepared in deionized water (100 mL). Circa 0.1 g of the catalyst was then dispersed in the solution, and the solution was purged with nitrogen gas for 30 min to remove dissolved oxygen. The reactor was irradiated by a 300 W xenon lamp (Newport, USA) equipped with a UV cutoff filter (*λ* > 420 nm) to simulate visible light irradiation. And the temperature was limited to below 30 °C by a water-circulating device.

The evolved hydrogen gas was collected and analyzed periodically by a Shimadzu GC-2014 gas chromatograph with a thermal conductivity detector (TCD), and the carrier was nitrogen. The HER activity was measured in µmol g^−1^ h^−1^, and the effects of catalyst loading, reaction temperature, and irradiation time on HER activity were examined.

Evolution of H_2_ and CO_2_ gases was measured on a Shimadzu GC–2014 instrument with a TCD detector. The calibration curves were constructed by the standard gas mixtures containing H_2_ and CO_2_ at known concentrations (50–500 µmol). The areas of the peaks were integrated and corrected against standards (*R*^2^ = 0.999 for H_2_, *R*^2^ = 0.998 for CO_2_). “The molar ratio of evolved gases is in good agreement with the stoichiometric relationship (H_2_ : CO_2_ ≈ 1.00 : 1.02) of formic acid dehydrogenation. Calibration plots (not presented) confirmed the linearity and accuracy of GC–TCD response for reliable quantification in the indicated range.

The apparent quantum yield (AQY) is a key parameter for evaluating the efficiency of photon utilization. AQY could not be determined in the present study, but it will be calculated in subsequent work on ferrioxalate actinometry by measurement of the photon flux angle, defined as:4
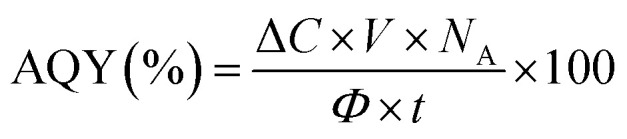
where Δ*C* is the change in concentration of reactant (mol L^−1^), *V* is the volume of solution (L), *N*_A_ Avogadro's number, *Φ* the photon flux (photons s^−1^), and *t* irradiation time(s). This value will provide a direct comparison of the photocatalytic efficiency with literature systems.

## 3. Results and discussion

### 3.1 UV-visible spectroscopy analysis

**Fig. 1:**
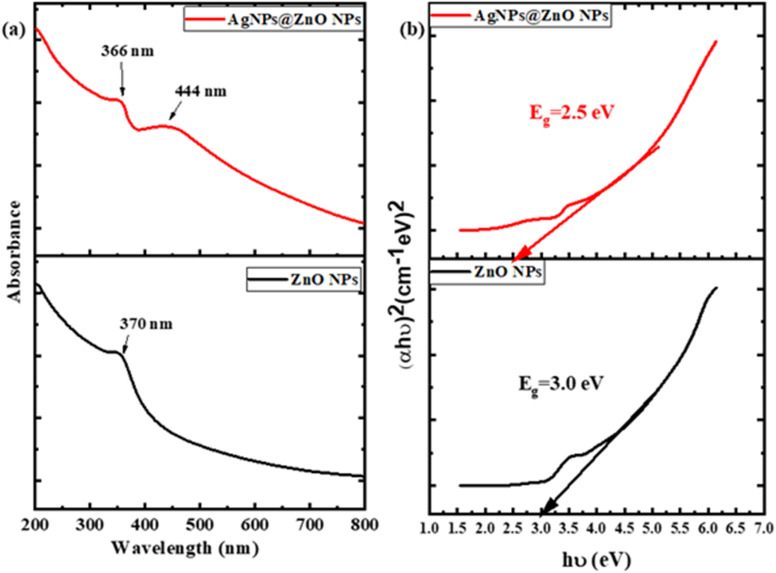
(a). UV-vis spectra of ZnONPs and AgNPs@ZnONPs exhibit redshift and Ag SPR peak at 444 nm (b) Tauc plots of ZnO NPs and AgNPs@ZnO NPs.

The UV-visible absorption spectra at the wavelength of 200–800 nm were used to determine the optical properties of pristine ZnO NPs and Ag-decorated ZnO NCs (AgNPs@ZnO NPs). Fig. 1(a) shows a strong and narrow peak of the pure ZnO NPs spectrum at approximately 370 nm. This is a result of the inherent electronic transition of VB–CB in the ZnO, which is a direct band gap semiconductor. Such a sharp peak means that there are ZnO NPs, which are a part of the wurtzite crystal lattice. It means that the particles are at the nanometer level, as the band gap of the quantum confinement effect changes marginally. The steepness of the peak suggests the high crystallinity and purity of the made ZnO NPs. Conversely, the absorption spectrum of the AgNPs at ZnO NC shows that there are two absorption bands, and this implies that a hybrid material is established. The ZnO band edge is still credited with the first absorption peak at 366 nm, although a little blue shifted, as compared to the peak of the pure ZnO sample. This minor modification might be explained by the surface modification of ZnO NP with silver, and lead to the local electronic environment variation due to the interaction between metals and semiconductors. Such interfaces modify the energy levels or band structure of ZnO to some slight extent and may be a contributor to the shift of the absorption edge.

More significantly, the spectrum of AgNPs@ZnO has an additional and wider band of absorption at 444 nm. The SPR of silver NPs does not result in this peak in the spectrum of pure ZnO. The free electrons on the metal NP surface phenomenon that causes the electrons to vibrate in synchrony with the incident light is referred to as the SPR. The presence of metallic silver could be proven by the SPR band; this is clearly the fact that the ZnO NPs were surface decorated with Ag. It would tend to move the SPR band of Ag typically to the visible region; therefore, that would extend the light absorption of the composite to the UV to the visible. The increased light absorption spectrum of the AgNPs@ZnO NC is very useful in practice. The greater absorption by the visible light range enhances the capture of sunlight, which is good in the application of photocatalysis, whereby increased visible light activity can also lead to the degradation of pollutants. Further, Ag in turn can serve as an electron sink to enhance the effective separation of photo-generated electron–hole pairs, to facilitate the overall photo-catalysis. In the field of antibacterial applications, the synergic effect of the ZnO and Ag is realized in the promoted generation of ROS when exposed to light in order to achieve a great deal of bacteria reduction.

One of the most important characteristics that governs the electronic, photo-responsive characteristic of semiconductor materials is the optical band gap energy (Tauc plot). The band gap values of the synthesized ZnO NPs and silver-decorated zinc oxide NCs (AgNPs@ZnO NPs) were determined through the UV-visible absorbance and Tauc plot analysis. The Tauc method works in accordance with the equation (*αhν*)_2_ = *A*(*hn* − *E*_g_) derived from a direct band gap, where *α* is the coefficient of absorption, *hν* is the energy of the photon, *A* is a constant, and *E*_g_ is the optical band gap. The band gap was determined by plotting (*αhν*)_2_ against *hν* and extrapolating the linear part of the plot to the energy axis where (*αhν*)_2_ = 0. As indicated in Fig. 1(b), pure ZnO NPs Tauc plots (black curve) have a linear region that is interrupted with the energy axes at around 3.0 eV, which is a perfect match with well-reported band gap values of the bulk ZnO (∼3.2 eV). The minor reduction to 3.0 eV is explained by quantum confinement effects, surface defects, or lattice strain incurred during NPs formation. This means that this is a nano-crystalline and direct band gap material. Even greater evidence of high crystallinity and purity of the phases of the prepared ZnO NPs is indicated by the presence of a distinct linear region.

The red shift in the band gap is significantly evident in the Tauc plot of the Ag-decorated ZnO NCs (AgNPs@ZnO NPs) where the extrapolated band gap is 2.5 eV. This reduction of the band gap of approximately 0.5 e V indicates that the Ag NPs have a considerable change in the electronic structure of ZnO. Some mechanisms can explain this band gap narrowing. To begin with, Ag on ZnO surface causes localized states of energy at the ZnO band gap owing to the electronic interaction between the two surfaces: metal–semiconductor interface. Secondly, Ag NPs have the SPR effect that increases light-matter coupling, which ultimately results in a large red shift of the absorption edge in the visible light region. Third, it is possible that silver acts as an electron sink where separation and charge transfer facilitation can occur, and therefore reduces the energy required to make the electron transitions. This reduction in the band gap enhances the energy gap of the material and leads to solar absorption enhancement, which is a vital property in applications such as visible-light photo-catalysis, antibacterial activity, solar power conversion, and optoelectronic materials. AgNPs@ZnO NCs possess a much superior photoactive performance than ZnO, owing to the amplified range of absorbance and improved efficiency of using photons. The flexibility in the optical band gap as a result of utilizing Ag not only confirms the establishment of an effective NC but also provides new opportunities for utilizing it in sustainable and light-driven technologies.

### 3.2 XRD analysis

**Fig. 2:**
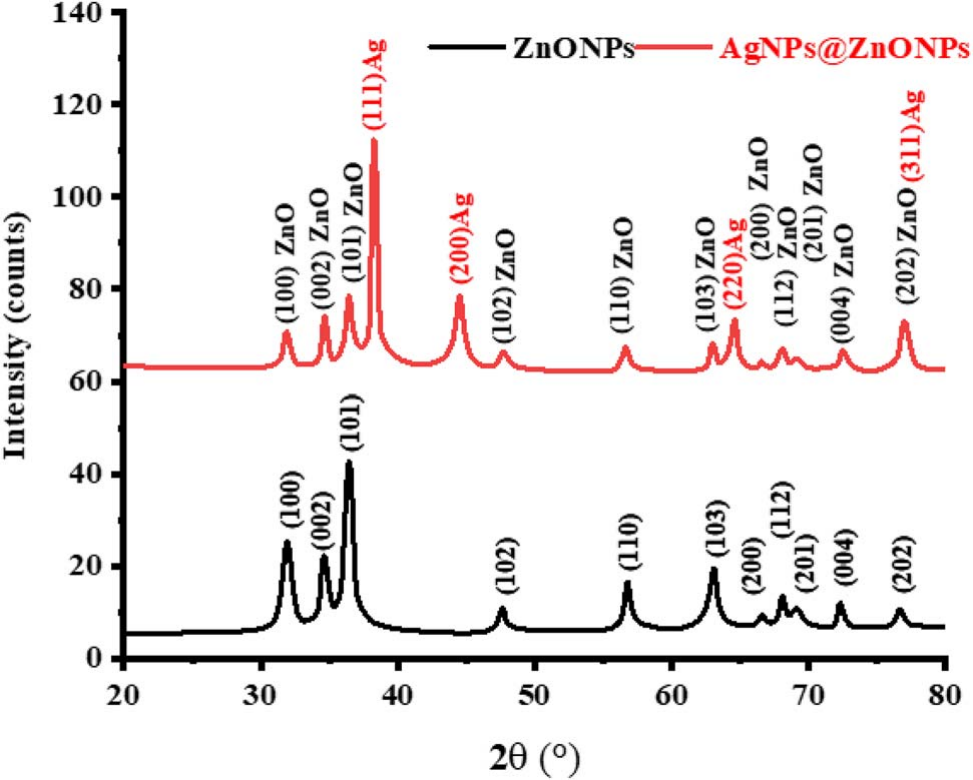
XRD patterns of the undoped ZnO (black) and Ag-doped ZnO NCs (red) showing the wurtzite phase of ZnO, and the presence of Ag metallic peaks.

The XRD analysis was used to investigate the crystal structure, phase purity, and the effect of silver doping on the ZnO NPs. Fig. 2 indicates the XRD patterns of the pure ZnO NPs (ZnO NPs) and Ag-decorated ZnO NCs (AgNPs@ZnO NPs). 56.6°, 62.8°, 66.4°, 72.6°, and 76.9° are the strong and narrow peaks in the XRD pattern of the ZnO NPs (black line). These peaks are attributed to the (100), (002), (101), (102), (110), (103), (200), (112), (201), (004), and (202) crystal planes, respectively. The peaks of diffraction are even compared with the standard data provided at the hexagonal wurtzite phase ZnO JCPDS card no. 36–1451. The presence of sharp and intense peaks is an indicator of good crystallinity of the ZnO sample, and the lack of any new or unexpected peaks shows that the sample is pure-phase ZnO with no secondary or amorphous impurities.

In the Ag-monolayer-decorated ZnO NC (AgNPs ZnO NPs), the primary reflection peaks of ZnO are present in the red XRD pattern, and all the peaks of the diffraction pattern in the XRD pattern can be ascribed to the hexagonal phase of ZnO, which points out that the crystal facet of ZnO core has not been destroyed by the introduction of silver. Of interest, additional peaks at 38.1°, 44.3°, 64.4°, and 77.5° would also be observed that can be indexed to (111), (200), (220), and (311) planes of face-centered cubic (fcc) silver as per JCPDS card no. 04-0783. The peaks are not coincidental at all, and they are not overlapping with the ones of ZnO, which means that the metallic silver NPs were indeed formed in the matrix. Fig. 2 shows XRD of ZnO–Ag NPs, having both ZnO and Ag peaks without significant movement of the Ag peaks towards the position of ZnO, indicating that the silver may not be doped into the lattice of the ZnO, but would form a second crystalline phase and would be deposited as silver on the surface of the ZnO NPs.

It is also in line with the explanation that Ag deposited on ZnO does not replace Zn^2+^ ions but rather is adsorbed on its surface. The presence of ZnO and Ag phases in the XRD pattern reveals that the bimetallic or hybrid NC possesses the characteristics of both ingredients. The decoration of ZnO with Ag does not deteriorate the quality of the crystal; however, it introduces an impurity phase that could enhance the optical and photo-catalytic characteristics of the material. The sharpness of the Ag peaks indicates that the silver nanoparticles are also crystalline and close-packed, which is very crucial for plasmonic action. Apart from the plasmon-resonance effect, the presence of Ag over the ZnO surface would improve electron mobility, decrease the recombination rate of electron–hole pairs, and enhance visible light photo-catalytic and bactericidal activity. This is mainly attributed to the LSPR effect and efficient charge carriers separation at the metal–semiconductor interface.

The mean size of ZnO and Ag phases could also be estimated using the Debye–Scherrer equation (DS):5
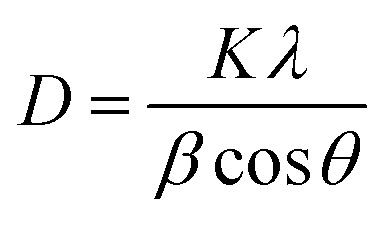
where *D* is the crystallite size, *K* is the shape factor (0.9), *λ* is the wavelength of Cu Kα radiation (1.5406 Å), *β* is the full width at half maximum (FWHM) in radians, and *θ* is Bragg's angle.^44^ Crystalite size calculations for ZnONPs and AgNPs@ ZnONPs are presented in Table 1.

**Table 1:** Crystalite size calculation for ZnONPs and AgNPs@ ZnONPs

Sample	2*Θ* (°)	*Θ* (°)	*β* (°)	*β* (radians)	*D* (nm)
ZnONPs	36.3	18.15	0.35	0.00611	23.9
AgNPs@ ZnONPs	38.1	19.05	0.32	0.00558	26.3

### 3.3 FTIR analysis of ZnONPs and AgNPs@ZnONPs

This is in line with the explanation that Ag is not deposited to replace the Zn^2+^ ions in the ZnO but deposits itself on it. The XRD pattern containing both ZnO and Ag phases verifies the appearance of a bimetallic or hybrid NC with the retention of the typical properties of each of the components. Silver decoration of ZnO does not ruin the crystal properties of ZnO but introduces an impurity phase, which can potentially enhance the optical and photo-catalytic characteristics of the material. The tight crystalline structure of the silver particles is also very critical to plasmonic functionality, as the Ag peaks are very narrow. Along with the plasmon-resonance effect, the presence of Ag on the ZnO surface will be able to increase the flow of electrons, slow the recombination rate among electron holes, and improve the effect of photo-catalyzing visible light and the bactericidal ability. This has mostly been attributed to the localized surface plasmon resonance (LSPR) effect and to the favorable separation of charge carriers at the metal–semiconductor interface.

**Fig. 3:**
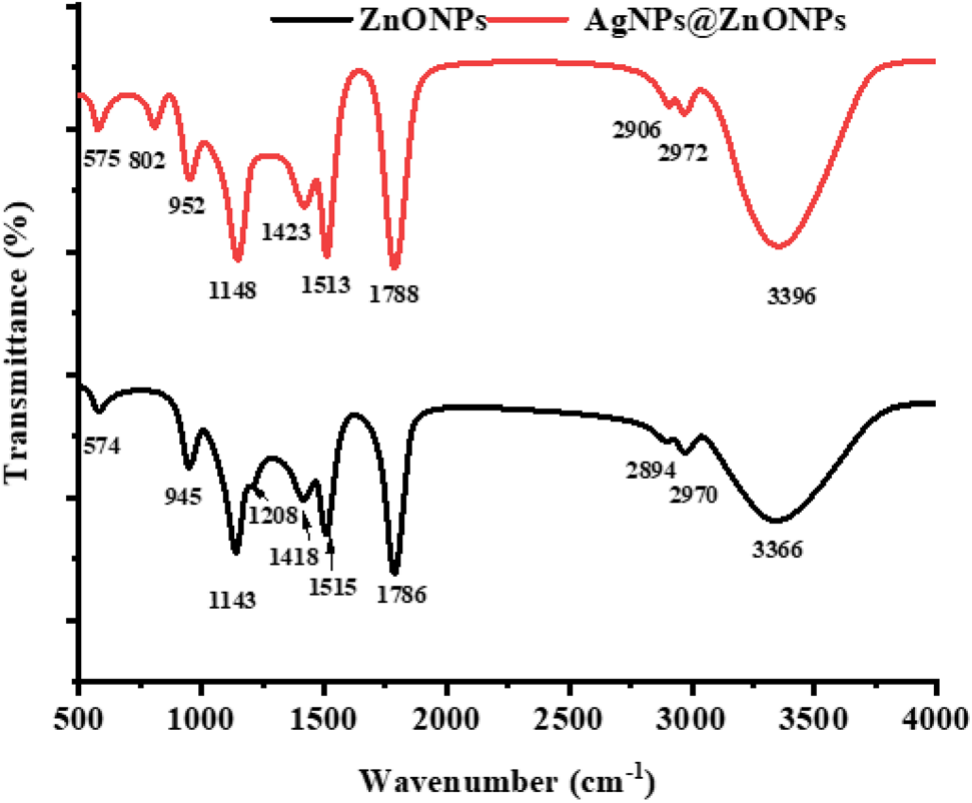
FT-IR spectra of ZnONPs and AgNPs@ZnONPs that demonstrate successful surface modification and loading of silver NPs.

FTIR spectra of synthesized ZnONPs and AgNPs @ ZnONPs in Fig. 3 reveal the existence of several functional groups and confirm the surface modification of ZnONPs with AgNPs. The band corresponding to O–H vibrations, in the ZnONPs (black curve), is 3366 cm ^−1^. This band is attributed to the presence of surface adsorbed moisture or hydroxyl groups on the surface of the metal oxide NPs, as they have high surface energy. These peaks at 2970 cm^−1^ and 2894 cm^−1^ are assigned to C–H stretching vibrations and show the role of organic capping agents and/or the rest of the plant-based biomolecules in the synthesis, especially when the synthesis route followed green route.

In the fingerprint (1800–500 cm^−1^) region, several well-defined bands can be observed. The peak of absorption at 1786 cm^−1^ corresponds to the stretching vibration of C=O (chemical carboxylic acid or ester), the bands at 1515 cm^−1^ and 1418 cm^−1^ are attributed to the C=C stretching vibration of the aromatic systems, which indicates the occurrence of aromatic compounds. This is possibly because of the presence of the compounds that are phytochemicals, which are reducing/stabilizing agents. The C=O stretching vibration was attributed to the absorption at 1208 cm^−1^ and 1143 cm^−1^, meaning that there was an alcoholic or ether moiety. The bands at 945 cm^−1^ and 574 cm^−1^ due to the Zn–O bond stretching are also quite important to observe, as it testifies to the creation of zinc oxide NPs. Contrary to this, the FTIR spectrum of AgNPs at ZnONPs (red curve) has the corresponding absorption bands with differences and changes in the peak intensity, which vividly demonstrates the successful interaction and modification of the surface of ZnONPs with AgNPs. The broad peak at 3396 cm^−1^ is also caused by O–H stretching. Still, it is slightly displaced compared to the one recorded when adding AgNPs, and the corresponding change in the hydrogen-bonding environment is observed. The C–H stretching at 2972 and 2906 (cm^−1^) is responsible for the presence of the peaks and shows that organic molecules remain on the surface.

Also, C=O and C=C vibrations were more visible with sharp and red-shifted bands at 1788 cm^−1^, 1513 cm^−1^, and 1423 cm^−1^ in the AgNPs at ZnONPs spectrum. This bathochromic shift and the amplification of the maximum intensities of these functional groups confirm that the electronic environment of functional groups after loading AgNPs had changed. In addition, the peaks of about 1148 cm^−1^, 952 cm^−1^, 802 cm^−1^, and 575 cm^−1^ C–O and metal–oxygen (Zn–O and Ag–O) vibration modes are additional evidence of the coexistence of the ZnO nanorod and Ag NPs in the NC. Apparently, the displacement of the position and strength of the peaks of ZnONPs to that of AgNPs@ZnONPs was the sign of successful silvery NP surface-functionalization of ZnONPs. The presence of hydroxyl, carbonyl, and aromatic functions was also used to prove the involvement of these biomolecules in the reduction and stabilization of NPs, particularly in green synthesis techniques. The dispersion stability, as well as the potential biological behavior of the NC material, is also caused by these active groups.

### 3.4 Surface morphology analysis by SEM

**Fig. 4:**
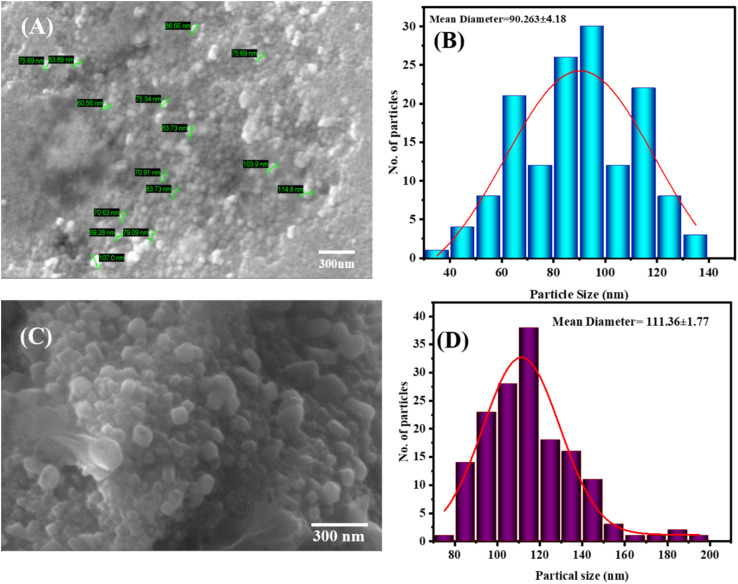
SEM images indicating surface morphology (A) ZnONPs (B), morphological analysis of ZnONPs (C), SEM image of Ag@ZnO (D), morphological analysis of Ag@ZnONPs.

SEM micrographs of ZnO and AgNPs carrier ZnO were quantitatively measured using the ImageJ software, where each sample had about 150 randomly selected particles measured to guarantee statistical reliability on surface morphology. SEM image and its size distribution histogram are displayed in Fig. 4A and B, while the SEM image of Ag@ZnO and its size distribution histogram are presented in Fig. 4C and D. The purified ZnO nanoparticles are quasi-spherical and irregular granular in morphology with significant agglomeration, which is represented by compact cluster-like formation owing to high surface energy and inter-particle interactions that are characteristic of the green-synthesized oxides. The size distribution of the particles is primarily in the range of 70–115 nm, and the histogram analysis results in an average diameter of the particle to be 90.2 ± 4.18 nm. This is much larger than the crystallite size of the samples determined by XRD (∼23.9 nm), which proves that observed particles are polycrystalline aggregates with multiple smaller crystallites, but not single crystalline domains.

Conversely, the AgNPs@ZnO nanocomposite is rougher and more disordered, and the nanodomains of silver are smaller and well-spaced on the ZnO skeleton, which represents the achievement of silver deposition. The statistical analysis of 150 particles indicates a relatively smaller size distribution (∼111.36–1.17 nm) and a smaller mean diameter of the particles. The reduction in the average particle size is due to the better dispersion and prevention of excessive aggregation owing to the strong metal-support interaction between Ag and ZnO. This morphological change enhances accessibility of the surfaces and interfacial contact, which is consistent with the increased surface area and the augmented catalytic activity of the surfaces in catalyzing hydrogen evolution as well as the photocatalytic degradation of tetracycline (Fig. 4).

### 3.5 EDS analysis

**Fig. 5:**
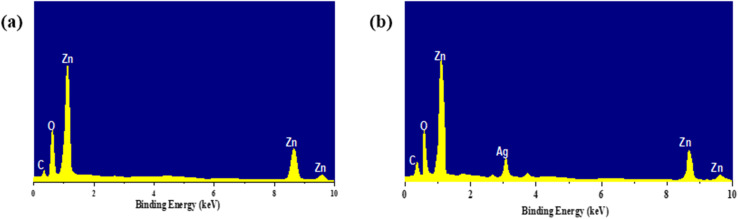
EDS spectra of pure ZnO (a), and Ag-decorated ZnO NCs (b), confirming the Ag addition and respective elements present in the samples.

The EDS spectra below show the elemental composition for the nano products synthesized and confirmation of the modified EDS surface composite. Fig. 5(a) is for the pure spectrum of ZnO NPs that shows intense peaks, and these are basically associated with Zn and O, which are the common constituents of ZnO. This large Zn peak at ∼1 keV and the other weak peaks for Zn 8–9 keV suggested that the concentration of Zn was much higher, and there is an oxygen peak at 0.5 keV, which also gives evidence of the presence of zinc oxide. The small spike of carbon (C) at a wave number of 1500 cm^−1^ is generally adventitious carbon, such as the contamination from the sample holder and air.

Fig. 5(b) displays the spectrum of silver decorated ZnO NC (AgNPsZnO). Except for the combined peaks of Zn and O in the pure ZnO sample, it has silver (Ag) peak at 3 keV. This peak affirms the presence of metallic silver at the ZnO surface. Ag signal and relatively stable Zn, O signals prove the absorption of the AgNPs@ZnO. The high and sharp Ag peak indicates that the Ag might be a surface decoration instead of substitution or doping, as no suppression to Zn/O could be observed. It is also in agreement with other characterization data, including work, UV-vis, and TEM, all indicating that Ag has been successfully anchored onto ZnO NPs, which at the same time depicts that the action of this material has been augmented and thus can be used in other applications, for example, photo-catalysis and antimicrobial activity.

### 3.6 TGA and nitrogen adsorption–desorption isotherm analysis for ZnONPs and AgNPs@ZnONPs

**Fig. 6:**
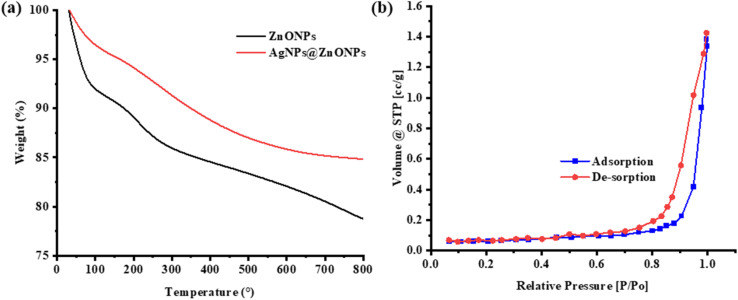
(a) TGA curves of ZnO NP's (black color) and Ag decorated ZnO NCs with red dotted lines representing weight percentage loss, depicting excellent thermostability in AgNPs@ZnO (b) nitrogen adsorption–desorption isotherm profile showing a type IV for the former with H3 hysteresis loop due to mesopores exhibiting slit shape Typical N_2_ physisorption was determined by using a physical absorption system.

The study of thermal stability and decomposition of ZnONPs and AgNPs@ZnONPs were studied by using TGA, which is represented in Fig. 6(a). The TGA was used to evaluate the thermal stability and decomposition of ZnONPs and AgNPs@ZnONPs, as illustrated in Fig. 6(a). The weight loss percentage (% by mass) in temperature (°C) as a result of programmed heating in air or an inert atmosphere is recorded. The ZnONPs (black curve) exhibit a two-step weight loss pattern. The adsorbed water and/or volatile organic compounds (*e.g.*, residual solvents or water present due to the synthesis) are associated with a first degradation step, which is below 150 °C. This is a common feature of all nano materials as a result of the large BET surface area, leading to surface hydroxyl groups. The second step in weight loss occurring at 150–800 °C may be explained well as thermal decomposition of any organic/capping agent which is used in its synthesis or plant-based phytochemicals (in the case of green route synthetic process). The ZnONPs could account for about 15% of the weight loss at 800 °C, indicating that there is less volatile or decomposable content in the NPs.

Compared with that, the AgNPs@ZnONPs (red curve) are thermally more stable and give a lower value of weight loss than pure PAN in the whole temperature range. The initial weight loss below 150 °C decreases slightly, which indicates there is a small decrease in the amount of physically adsorbed water (moisture) content. The outgassing of weight in the temperature range from 150 to 800 °C is also less than that of ZnONPs. The % wt loss is near to be 12% for AgNPs@ZnONPs, and thus, the presence of silver NPs is responsible for improving the thermal stability of NC. This enhancement may be attributed to much contact between the Ag particles and the ZnO, and some oxygen volatiles accepted on the surface being reduced and stabilized in a metal/semiconductor framework. Higher thermal stability of AgNPs@ZnONPs confirms that decoration with silver modifies not only surface chemistry but also improves the structural stability of NC. The second feature is especially relevant when higher temperatures are involved, as in the case of catalysis, antimicrobial, or electronics coating subjected to thermal cycling.

The quantitative parameters of the specific surface area and porosity characteristics for ZnONPs and AgNPs@ZnONPs were measured from the BET method results collected *via* adsorption isotherm data (relative pressure (*P*/*P*_0_) 0.05–0.30). The adsorption–desorption isotherms (Fig. 6(b)) are characterized by a common type-IV profile with H_3_ hysteresis loop, which is due mainly to interparticle void spaces formed by agglomeration of nanoparticles rather than intrinsic microporosity. The BET surface area of the pristine ZnONPs was 18.6 m^2^ g^−1^ while it increased to 42.3 m^2^ g^−1^ for AgNPs@ZnONPs. The increase is due to the deposition of Ag nanoparticles, which promotes the dispersion of particles and inhibits the aggregation of ZnO, leading to more mesoporous interfaces available. The pore volume of AgNPs@ZnONps was 0.083 cm^3^ g^−1^, and the average pore diameter, ranging from ∼14–18 nm, is within the mesoporous range, thus confirming the hierarchical porous structure. Such enhancement in mass transfer of reactant species during photocatalytic and formic acid dehydrogenation reactions is attributed to a larger amount of available sites for tetracycline and formic acid molecules to be adsorbed on *via* the increase in surface area and mesoporosity. Therefore, the superb catalytic activity of AgNPs@ZnONPs can be ascribed to not only the Ag–ZnO interfacial charge transfer effect but also to heightened surface accessibility from its larger specific surface area, as evidenced by BET studies.

Morphology studies of the NPs performed by the SEM and transmission electron microscopy revealed the NP to be uniform with colonies of pores. These structural components are advantageous for operational improvement. The mesoporosity is beneficial for drug delivery with good adsorption and release of drugs. For example, in the field of photo-catalysis, it is beneficial to have a larger specific surface area and interconnected pores for light absorption and mass transfer, as well as improved photo-catalytic and antimicrobial activity, which should proportionate with the increasing number of active ROS-producing sites due to silver NPs, like AgNPs@ZnONPs, which was observed. Both of them and the former reveal that porous nanostructure, as a multifunctional material, has an effective synthesis.

### 3.7 AgNPs@ZnO NCs for catalytic dehydrogenation of FA

Dehydrogenation of FA is the elementary step in catalytic hydrogen production, especially under conditions close to room temperature, where catalyst performance is crucial for the energy conversion efficiency.^4^ The HER performance of AgNPs, ZnO NPs, and their NC AgNPs@ZnO in the present work was carefully studied to investigate their role as a renewable and economical catalytic system. Several experiments were conducted to assess the effect of the key reaction factors, *i.e.*, solvent, catalyst loading, reaction time, pH, catalyst composition ratio, temperature, and *E*_a_ on the rate of hydrogen production. The performances of these catalysts have been studied by determining the cumulative volume of evolved hydrogen gas, by TOF, and kinetic measurements, which yield important information about catalytic mechanisms and efficiencies under different experimental conditions. It is concluded from the results that AgNPs@ZnO NCs possess remarkably improved HER activity compared with ZnO, which is ascribed to the synergistic effect of Ag and ZnO that promotes the charge separation, light harvesting, and surface reactivity. The comprehensive characterization verifies the potential of AgNPs@ZnO as a bi-functional electrocatalyst toward sustainable energy.

#### 3.7.1. Influence of composition, catalyst loading, and solvent on FA degradation

In order to better investigate the role of various parameters on efficient FA dehydrogenation on the as-prepared catalyst, we conducted a series of systematic studies by considering the effect of support material, catalyst loading, and solvent on the production of H_2_ gas.

**Fig. 7:**
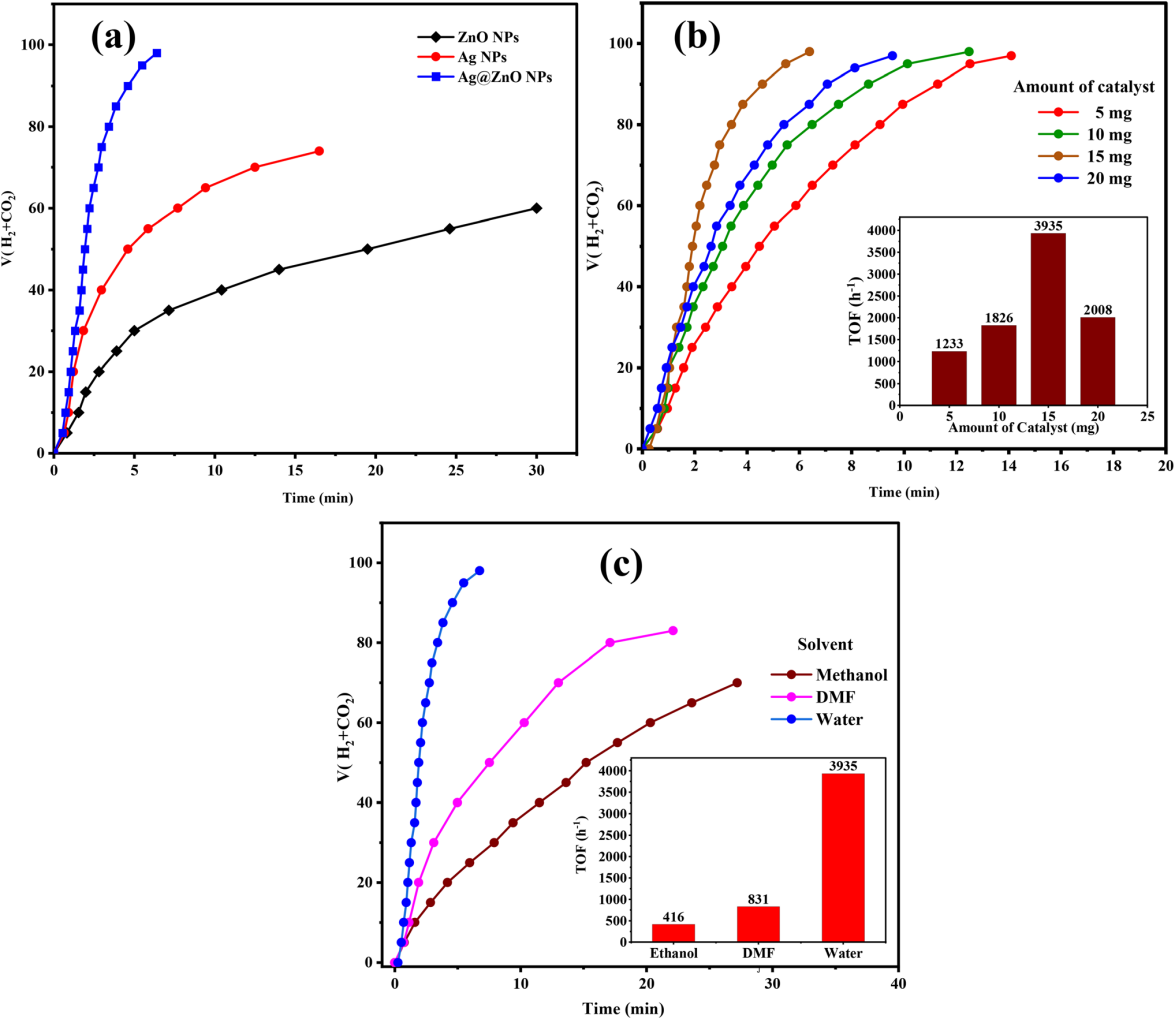
(a) The plots of evolved gas (CO_2_ + H_2_) against time for composition of the catalyst (Ag NPs, ZnO NPs, and Ag@ZnO NCs), (b) the plots of evolved gas (CO_2_ + H_2_) *versus* time at various loadings (5 mg, 10 mg, 15 mg, 20 mg) and corresponding TOF values, and (c) HER performance of the catalysts in various solvents (methanol, DMF, water) and corresponding TOF values for using Ag@ZnO NCs at pH 4 and temperature 343 K.

The comparison of the catalytic efficiencies of ZnO NPs, Ag NPs, and Ag@ZnO NCs demonstrates the key role of SI (ZnO) in effectively improving the catalytic performance. Pure ZnO NPs were the least active (see Fig. 7(a)) and evolved ∼60 mL of the gas in 30 min, reflecting their poor inherent ability to drive FA dehydrogenation. While pristine Ag NPs showed comparatively higher activity (evolving 70 mL of the gas in 15 min), arising from their well-known metallic catalytic feature, they were surpassed by Ag@ZnO NCs that rapidly generated nearly 100 mL of gas within only 6.4 min. This remarkable activity arises from the special role of ZnO, which acts as a support with a high surface area able to favor uniform dispersion of Ag nanoparticles and prevent their aggregation, and a modifier for the electronic structure of Ag *via* strong metal-support interaction. The amphoteric properties of ZnO further generate hydroxylated sites under reaction conditions that work synergistically with Ag active centers for FA adsorption and activation. In addition, the Ag–ZnO interfaces act as heterojunctions so as to benefit from charge transfer and subsequently facilitate the scission of C–H and O–H bonds in FA. Therefore, the enhanced catalytic performance of Ag@ZnO compared to that of each part (Ag or ZnO) in metal/support could demonstrate a synergistic effect between ZnO support and supported metal for superior FA dehydrogenation activity.

The effect of catalyst loading on FA dehydrogenation onto Ag@ZnO NCs was further investigated, and it was found that activity initially improved with the increase in the amount of catalyst until reaching an optimum value, followed by a decrease (Fig. 7(b)). The 95 mL of gas was generated with a low dosage at 5 mg, and the TOF value was 1233 h^−1^, which is due to a smaller number of active sites. When the catalyst amount was increased to 10 mg and 15 mg, the activity of those enhanced dramatically, especially for 15 mg sample, with a maximum TOF (3935 h^−1^), and the fastest gas evolution curve was achieved as a result of maximized active positions availability and improved FA adsorption. However, increasing the loading to 20 mg reduced the activity (TOF value 2008 h^−1^) as a result of particle aggregation due to aggressive catalyst loading, mass transport limitations, and poor dispersion in the reaction medium. Consequently, the most suitable catalyst dosage of 15 mg leads to a tradeoff among exposing a large surface area, using more active sites, and achieving some reaction kinetics because other lower or higher dosages are not conducive to the catalytic performance.

The solvent significantly determines the catalytic performance of Ag@ZnO NCs on FA dehydrogenation, as clearly shown by the varied TOF values in water, DMF, and methanol media. The catalyst also exhibited the best activity in water with a TOF of 3935 h^−1^, suggesting that FA is more compatible with water to be used as a solvent. Water becomes a polar medium, helping to stabilize the charged intermediates, formate species, and, in addition, it increases the H^+^ availability by lowering their energy barrier for H_2_ and CO_2_ formation. On the other hand, the value for TOF obtained with DMF was much smaller (831 h^−1^), which is related to its aprotic character, and consequently restricts H^+^ engagement and hence is not favourable for effective dehydrogenation. Although DMF might stabilize the FA molecule to some extent, its lower hydrogen bonding ability relative to water would affect the facile breaking of C–H and O–H bonds during the decomposition of FA. Methanol, however, showed the lowest activity with a TOF of 416 h^−1^. This poor performance is likely due to the competitive adsorption of methanol molecules on the surface of the catalyst, which could block the active Ag–ZnO sites and also interfere with FA adsorption. Moreover, the protic but relatively less polar character of methanol, compared to water, reduces its capacity to stabilize the abstraction intermediates leading to dehydrogenation. These results unequivocally indicate that water is the best solvent not only regarding FA activation and intermediate stabilization, but also due to its so far unappreciated capacity of transporting protons, which are paramount in promoting high catalytic turnover for hydrogen generation.

#### 3.7.2. Influence of reaction parameters on FA degradation using AgNPs@ZnO NCs

The performance of Ag@ZnO NCs for the HER action was carried out under different pH values, ratios of FA/SF, and reaction temperatures. The purpose is to investigate the influence of these factors on the rate of generation of hydrogen gas, as well as to establish the optimal operation conditions for minimum losses.

**Fig. 8:**
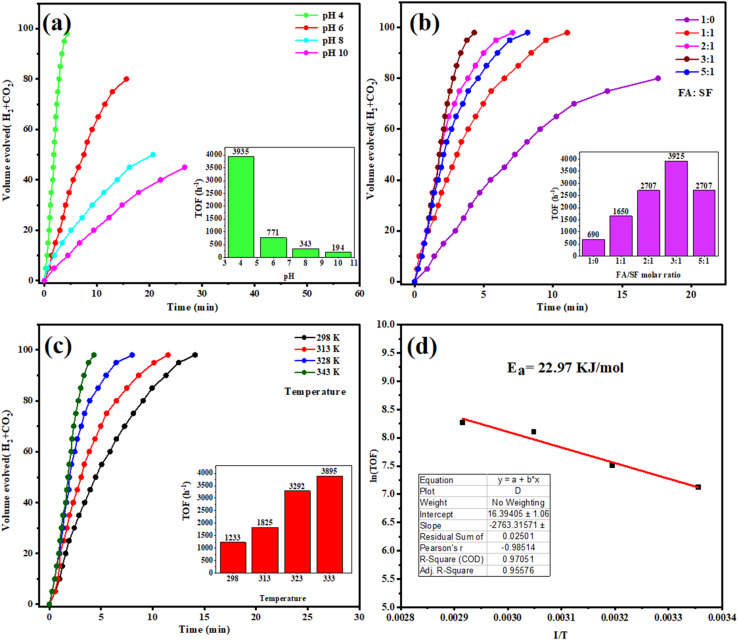
(a) The changes in HER rate at different pH values (pH 4–pH 10). (b) The volume of the generated hydrogen based on different FASF ratios during the HER reaction. (c) Hydrogen evolution rate at different temperatures (298 K–343 K). (d) Arrhenius plot used for the determination of the *E*_a_ for the catalytic FA dehydrogenation.

As shown in Fig. 8(a), the influence of pH on hydrogen evolution activity has been examined using different pH levels (range: 4–10) for the reaction medium. The data showed that the catalytic dehydrogenation of FA on Ag@ZnO NCs was highly pH dependent; the activity decreased with increasing pH from 4 to ∼pH 10. At pH 4, the catalyst showed an enhanced activity and maximum TOF 3935 h^−1^, releasing 100 mL gas within 6.4 min compared to those 771, 343, and 194 h^−1^ at pH 6, 8, and 10, respectively. The excellent activities at pH 4 are associated with the abundant demand of protons, leading to FA being mainly in molecular form (HCOOH) rather than as ions of formate, and thus reducing the activation energy to perform the dehydrogenation process faster. Additionally, the proton-rich environment promotes the adsorption of FA on Ag sites and stabilizes reaction intermediates; amphoteric ZnO support co-acts with Ag NPs to facilitate charge transfer and catalyze C–H and O–H bond activation at the interface synergistically. In addition, an acidic environment also inhibits other pathways, such as CO formation, which may compete with hydrogen production and becomes more pronounced at alkaline conditions dominated by formate ions, resulting in lower kinetics and low productivity of hydrogen. These factors collectively account for the fact that the acidic condition (pH 4) provides the optimal conditions for FA dehydrogenation, so Ag@ZnO NCs are very efficient catalysts for hydrogen production in a slightly acidic solution.

In addition, the catalytic dehydrogenation of FA over Ag@ZnO NCs was studied, and results indicate that the molar ratio of FA to SF (Sodium Formate) played a crucial role in promoting hydrogen generation performance. As illustrated in Fig. 8(b), 1 : 0 exhibited the lowest TOF value of 690 h-1, which is accompanied by a sluggish gas-evolving profile, suggesting poor stability and kinetics control over pure FA decomposition. The activity is much enhanced when SF is added, with TOFs 1650, 2707, and a maximum of 3925 h^−1^ for FA/SF ratios at 1 : 1, 2 : 1 and 3 : 1, respectively, showing a faster and step rise for gas volume (H_2_ + CO_2_) *vs.* time profile. Such a remarkable improvement could be attributed to the role of sodium formate, which strongly stabilizes the reaction medium and offers more formate anions as active intermediates, leading to facilitating the driving of the dehydrogenation pathway rather than undesirable CO formation. The best proportion with a 3 : 1 ratio is conducive to the synergistic effect between molecular FA and formate ions to produce more reactive species adsorbed on Ag active sites, such that it can promote bond cleavage at the interface of Ag–ZnO effectively. It should be noted that when the FA/SF ratio is increased to 5 : 1, the activity declines slightly (TOF 2707 h^−1^), indicating that too high a content of FA dissociates not only the stabilized effect of SF but also an appropriate reaction environment. Taken together, these results unequivocally show that the introduction of sodium formate is crucial to adjust the catalytic activity, and FA/SF in a ratio of 3 : 1 provides an optimal condition for fast, selective, and highly efficient hydrogen production on Ag@ZnO NCs.

The impact of temperature on HER is shown in Fig. 8(c), which we performed at various temperatures (298 K, 313 K, 323 K, and 343 K). The hydrogen production rate rose with the temperature, suggesting a thermally activated catalytic process. At higher temperatures, energy is supplied to break the kinetic barriers for the charge carrier mobility and reaction kinetics. This temperature-dependent nature of concentration, in the case of Ag@ZnO, is especially useful because at higher temperatures, efficient electron transfer paths from the AgNPs to ZnO are improved. The increased thermal energy also reduces the charge recombination, promotes desorption of reaction products, and promotes the fast hydrogen evolution. These results underscore the stability of the catalyst and the fact that it can work efficiently in a wide range of temperatures; it seems to be compatible with low and moderate-thermal-input systems.

For understanding the energy barrier toward HER, the *E*_a_ (*E*_a_) was determined from the Arrhenius equation and is illustrated in Fig. 8(d). The Arrhenius plot (ln *k* against 1/*T*) is linear, and the calculated *E*_a_ = 22.97 kJ mol^−1^ for the Ag@ZnO catalyst system. This low value implies that the FA dehydrogenation of FA on Ag@ZnO is thermodynamically favorable, and the energy required for the reaction is very low. The composite had a relatively lower *E*_a_ compared with the pure AgNPs or ZnO NPs, suggesting that the bimetallic/semiconductor interface can enhance the charge separation and make the reaction process more rapid. The addition of AgNPs to enhance light harvesting and the surface plasmon resonance, and ZnO's stability and high surface activity, all play an important role in overcoming this low activation barrier. These results further confirm that Ag@ZnO is an efficient hydrogen evolution material and thermodynamically competitive, suggesting potential applicability of Ag@ZnO in low-temperature hydrogen production devices.

A control experiment was performed in pure water under identical conditions but in the absence of formic acid. Negligible hydrogen evolution was observed, confirming that the high HER performance originates from the catalytic dehydrogenation of formic acid rather than water splitting. The corresponding control data have been added to the revised manuscript as Fig. S1 in the SI for clarity and completeness.

#### 3.7.3. GC-TCD analysis of gaseous products

**Fig. 9:**
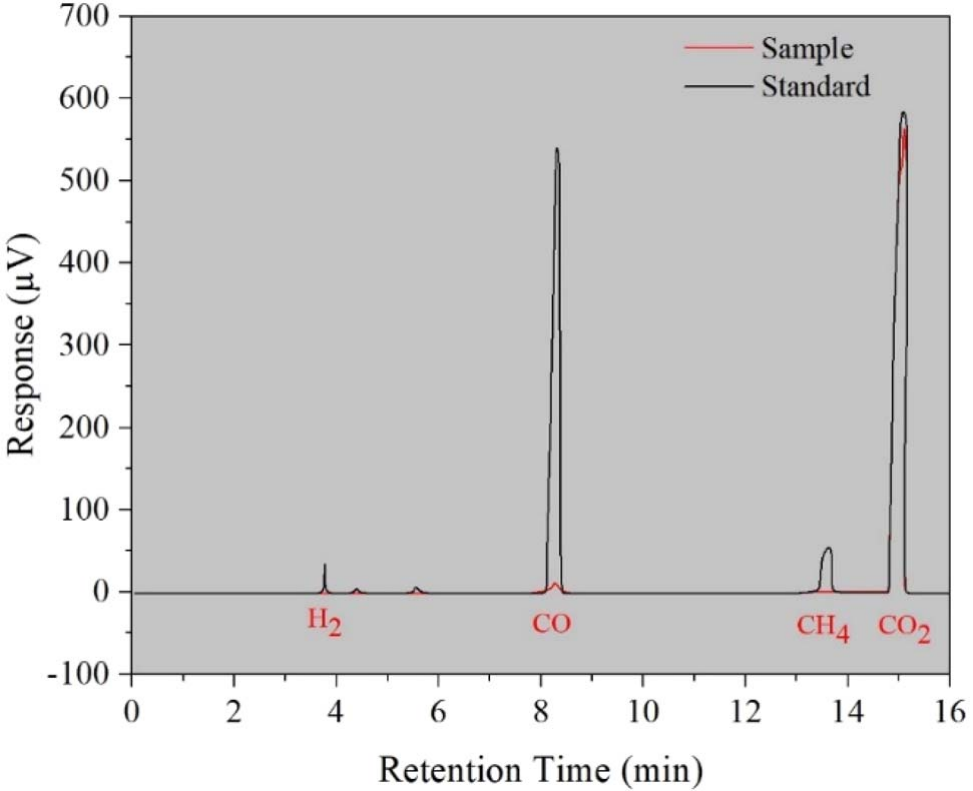
GCTD analysis verifies selective H_2_ and CO_2_ evolution without CO formation during HER.

To analyze the gaseous products evolved in the reaction of optimized AgNPs@ZnO, Gas Chromatography with Thermal Detector (GCTD) was carried out. In Fig. 9, the chromatogram displays a relative profile of the standard gas mixture (black line) and actually evolved gases of the reaction (red line). In the normal gas trace, there are separate peaks for H_2_ (∼4 min), CO (∼8 min), CH_4_ (∼13.5 min), and CO_2_ (∼15.5 min), acting as markers of identification. However, the experimental sample shows large peaks at H_2_ and CO_2_ only, and no measurable peak is found at the CO retention time, corroborating the lack of carbon monoxide.

This outcome emphasizes the selective dehydrogenation activity of the AgNPs@ZnO photo catalyst, wherein the organic substrate gets fully oxidized, yielding only hydrogen and carbon dioxide, with no CO or other toxic gaseous intermediates. The lack of CO conclusively suggests the clean and efficient oxidation route provided by the catalyst. These results, therefore, substantiate the use of AgNPs@ZnO as a very selective and eco-friendly catalyst for hydrogen evolution from the sun, substantiating its candidacy for sustainable energy applications. Fig. S2 in SI shows GC-TCD analyses for standard and sample evolved gases separately.

#### 3.7.4. GCTD-based mechanistic insight into FA dehydrogenation over AgNPs@ZnO nanocatalyst

**Fig. 10:**
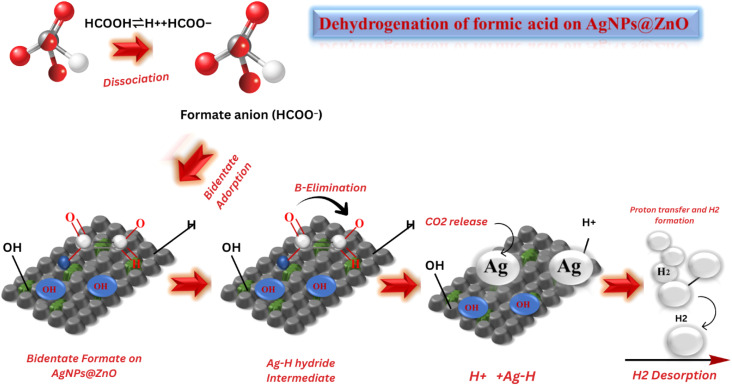
GCTD-based schematic representation of the FA dehydrogenation pathway over AgNPs@ZnO nanocatalyst showing desorption, intermediate formation, and H_2_ evolution.

The dehydrogenation of HCOOH on AgNPs@ZnO NC-based catalyst proceeds *via* a typical surface-mediated reaction mechanism and involves a series of adsorption, activation, bond breaking, and desorption steps governing the favorable hydrogen evolution under mild conditions as shown in Fig. 10. In water, the initial step of dissociation of FA occurs, resulting in the generation of the proton (H^+^) and formate anion (HCOO^−^). The dehydrogenation of FAA to FA on AgNPs is mostly mediated *via* the formate anion, which is initially adsorbed on the surface of AgNPs@ZnO catalyst. The ZnO surface, which is abundant in hydroxyl and oxygen vacancy sites, serves as a high-surface-area support with the ability to confine and stabilize the formate species *via* electrostatic interactions. At the same time, the well-dispersed AgNPs on the surface of ZnO act as the highly active catalytic sites and electron mediators. The formate anion first binds adsorbed in a bidentate form, which in turn accommodates a monodentate configuration during structural rearrangement. Such transformation is indispensable because in the bidentate binding mode, the reactive C–H bond is located too far away from the catalytic surface to enable efficient cleavage, whereas the monodentate configuration orients the C–H bond closer to the AgNPs' surface, leading to an effectively activated C–H bond.

In this monodentate conformation, the β-H elimination pathway becomes energetically feasible. This leads to cleavage of the C–H bond assisted by the surface electrons of the AgNPs, and CO_2_ is released to produce a metal–hydride intermediate (Ag–H). Concurrently, the surface adsorbed protons (H^+^), formed *via* the dissociation of FA, react with the hydride species to form molecular hydrogen (H_2_) that desorbs from the surface, closing the catalytic cycle. The corresponding total reaction, HCOOH → H_2_ + CO_2_, selectively occurs with low CO production, a known poison for catalysts. The synergistic effect between Ag and ZnO is the key during the whole process: the AgNPs could facilitate the transfer of electrons and provide an effective pathway with a low barrier for C–H activation, while the ZnO could serve as a strong platform to support the formate species and stabilize the intermediates.

Additionally, the small value of *E*_a_ (≈37.34 kJ mol^−1^) deduced from the Arrhenius plot further indicates the high efficiency of the AgNPs@ZnO system for HER at low temperature. The NC microstructure promotes efficient charge separation and transport, which suppresses hk-electron recombination and enhances the total concentration of active species on the surface. This mechanistic path unveils not only the function of each unit block within the MOF composite but also the importance of catalyst architecture in dictating the kinetics and thermodynamics of FA dehydrogenation. Thus, AgNPs@ZnO is a potential green catalyst for hydrogen production, and a sustainable, efficient, and low-temperature process for H_2_ generation from a renewable chemical hydrogen carrier is proposed.

#### 3.7.5. Post-catalysis characterization

To examine the stability of AgNPs@ZnO after HER, post-catalytic PXRD, FTIR, and SEM analysis were performed, as shown in Fig. S3. The PXRD pattern of the used catalyst retains the characteristic diffraction peaks of ZnO, confirming preservation of the crystalline structure. In addition, weak reflections corresponding to Ag_2_O are observed, indicating partial surface oxidation of Ag nanoparticles during the catalytic process. This oxidation appears to be limited to the surface and does not significantly affect the overall crystallinity of the composite.

The FTIR spectrum of the post-catalysis sample shows the characteristic Zn–O stretching vibration along with surface hydroxyl bands. Notably, additional bands observed around ∼1740 cm^−1^ and ∼1560 cm^−1^ can be attributed to C=O and COO^−^ stretching vibrations, respectively, indicating the adsorption of formate species on the catalyst surface during HER. These features confirm the interaction between reaction intermediates and the active surface sites. The presence of these bands suggests surface adsorption rather than permanent structural modification.

SEM analysis reveals that the overall morphology of the nanocomposite is maintained after catalysis, with only slight surface roughening and minimal nanoparticle aggregation observed. Collectively, these results demonstrate that AgNPs@ZnO preserves its structural integrity with minor surface oxidation and reversible adsorption of intermediates, confirming its stability under HER conditions.

### 3.8 Photo-catalytic degradation analysis

Photo-catalysis has been given much interest as an effective and environmentally friendly technique of eradicating organic contaminants in wastewater, such as antibiotics like TC that are resistant to traditional treatment procedures.^45–47^ AgNPs onto zinc oxide (AgNPs@ZnO) have been employed in this paper as a visible-light-driven photocatalyst for the photo degradation of TC under solar light. The charge separation efficiency and the light absorption spectrum of ZnO are enhanced by the presence of Ag, which results in an increment in the photoactivity.^48–50^ This paper presents a systematic study of the degradation mechanism, the nature of shifts in the UV-vis spectra over time, a kinetic model based on pseudo-first-order equations, calculations of Arrhenius-based *E*_a_ and thermodynamic parameters, and the impact of radical scavengers. AgNPs@ZnO has also been compared to the reported catalysts in terms of general photo-catalytic activity to emphasize its high catalytic activity and its usefulness.

#### 3.8.1. Photocatalytic degradation of TC using AgNPs@ZnO

The time-dependent UV-vis spectroscopy, thermal variation, kinetic modeling, and *E*_a_ analysis were used to examine the whole performance of AgNPs@ZnO NC in the degradation of TC under solar irradiation.^51,52^ These experiments explain the photo-physical response as well as the mechanism of degradation underlying which the catalyst has a very good potential of being used in solar-assisted wastewater treatment.^53,54^

**Fig. 11:**
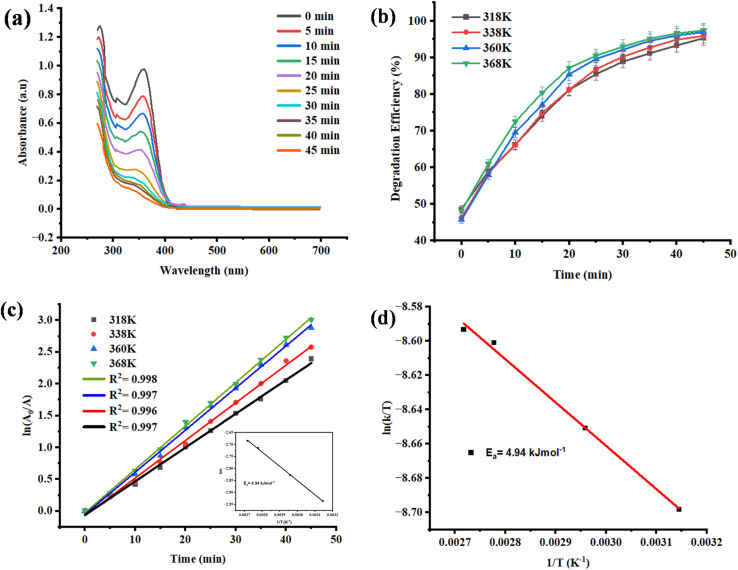
(a) UV-vis absorbance spectra of TC over AgNPs@ZnO under solar light; (b) the effect of temperature on decomposition rate: (c) pseudo-first-order kinetic equation with the Arrhenius plot inset and (d) linearity of the Arrhenius plot with ln *k* plotted *versus* 1/*T* as well as *E*_a_ at 4.94 kJ mol^−1^.

The UV-vis absorbance spectra of TC during photo-catalysis by the sunlight in different time intervals (0–45 minutes) are shown in Fig. 11(a). TC also shows good absorption in the range of 275 to 370 nm, which is primarily attributed to π–π* transitions in aromatic structure.^55^ When the solar exposure of the AgNPs on the surface of the ZnO was observed, a gradual and steady decrease in the intensity of the absorbance with time was recorded, which implies that an active degradation of the antibiotic molecule was taking place.^56^ This sudden drop can be explained by a high concentration of photo-generated charge carriers and open sites on the catalyst in the surface, which can be attacked by ROSs, including ˙OH and ˙O_2_^−^, quickly. The rate of degradation decreases gradually as the reaction progresses and active sites are saturated or covered with the intermediates, approaching a near-complete degradation (approaches 99 percent degradation) in the reaction in 45 minutes, indicating that the reaction is in equilibrium or is at or near saturation.^57,58^ This activity underpins the great performance and short-duration property of the NC in ambient photocatalytic reactions.

To determine how the photo-catalytic activity is affected by thermal energy, temperature-dependent degradation studies were done Fig. 11(b). The reactions were done at four temperatures, *i.e.*, 318 K, 338 K, 360 K, and 368 K. The degradation rate rose with temperature to a high of about 84, 89, 94, and 97 percent within 50 minutes. The increased activity of the processes at higher temperatures can be attributed to the increased active movement of the molecules, which facilitates the increased rate of collision of TC molecules and active radicals. Besides, elevated temperature could enhance the desorption of degraded products on the catalyst surface, hence retaining catalyst reactivity. The second factor is the fact that, with an increase in temperature, the separation and migration of photo-generated electron–hole pairs is enhanced, and this minimizes recombination and enhances the availability of radicals. This thermal-aided improvement reflects the thermal sensitivity of the AgNPsZnO photo catalyst and its applicability in practice at different conditions of the environment.

The kinetics of the reaction were also studied based on the pseudo-first-order kinetic model Fig. 11(c), in which the ln(*A*_0_/*A*_*t*_) was plotted against irradiation time at each temperature. The plots in Fig. 1 and 2 are linear, and the *R*^2^ values are greater than 0.996, which confirms that the degradation of TC over AgNPs@ZnO is pseudo-first-order in nature and the rate of the reaction is directly proportional to the concentration of TC molecules. This means that the rate-limiting stage would be the interaction between the pollutant and the photo-generated ROS on the catalyst surface. Their slopes gave the rate constants (*k*), which rose with temperature, as theoretically anticipated for thermally activated processes of Arrhenius-type behavior.

A quantitative analysis of the thermal dependence of the rate of the reaction was done by plotting ln *k versus* 1/*T* (Arrhenius plot), which is displayed in Fig. 11(d). The apparent *E*_a_ of the photocatalytic degradation process was then obtained with the Arrhenius equation using the linear slope of this plot. The calculated *E*_a_ value is 4.94 kJ mol^−1^, which means that the reaction has a low-energy barrier. Such a low *E*_a_ is very desirable because it means that the reaction needs minimal thermal input to be run efficiently, thus it can be used in solar-light-driven applications. This low energy requirement may be attributed to the fact that Ag NPs have been able to attach well to the ZnO matrix, providing a high surface area, and to the synergistic interaction between the Ag NPs and the ZnO matrix, which all lead to the minimization of the kinetic barriers of the reaction.

#### 3.8.2. Effect of initial pH and catalyst loading on photo-catalytic degradation efficiency of TC

**Fig. 12:**
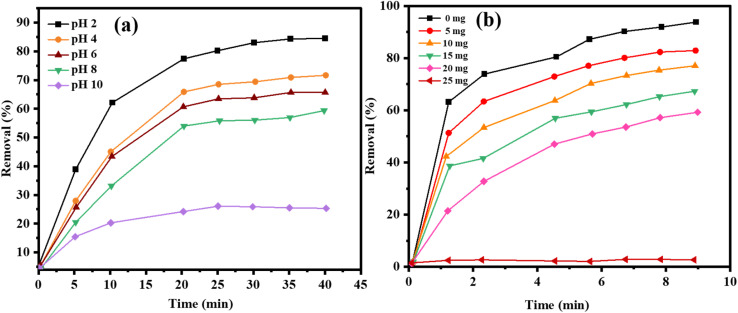
(a) Effect of the initial pH on the photo-catalytic degradation of TC by ZnO, AgNPs, and AgNPs@ZnO NCs under visible light (0–40 min). (b) Effect of the loading amount of catalyst on the photocatalytic degradation efficiency of TC by ZnO, AgNPs, and AgNPs@ZnO NCs at pH 4, TC concentration = 50 mg L^−1^ (0–40 min).

The initial pH of photo-catalytic reduction of TC was investigated carefully in relation to the initial pH under the catalysis of ZnO, AgNPs, and AgNPs/ZnO NCs. The pH of the TC solution also contributes greatly *via* its impact on the surface charge of the photo catalyst as well as the ionization status of the TC molecules, which in turn influences the kinetics of the degradation. A photo-catalytic experiment with visible light was performed with a TC concentration of 50 mg L^−1^ and a catalyst concentration of 40 mg at different pH levels between 2 and 10. Fig. 12(a) shows that the catalytic activity of all three catalysts varied naively with pH. In the case of ZnO, the maximum degradation efficiency was obtained at pH 6 (81%), and at pH 2 (58%) and pH 10 (39%), the degradation efficiency was significantly higher, indicating that suboptimal interactions were taking place at pH 6. Moderate rates of degradation were obtained at pH = 4 (74%) and pH = 8 (75%).

There was a moderate level of photo-catalytic activity of AgNPs in the reactors at different pH levels, reaching its peak in 74% degradation at pH 4. However, the level of degradation at pH was not very high: the degradation at alkaline pH (*e.g.*, pH 10) was about 30%, meaning that the stability of AgNPs and their capacity to generate ROS at higher pH values was limited. In contrast, AgNPs were found to exhibit greater and constant photo-catalytic activity across the entire pH range and 99 percent maximum photo-degradation at pH 4. This enhanced activity is attributed to ZnO Ag NPs synergy. Availability of Ag offers the segregation of the charge carriers and increases the range of light absorption, and ZnO is an effective oxidative hydroxyl radical formation location, which contributes to the breaking down process. AgNPs@ZnO remained a comparatively high degradation efficiency (68%) at pH 10, which was more than that of the other two catalysts. All catalysts showed reduced rates of degradation at basic pH (pH = 8–10). This could be explained by the suppression of OH radical evolution and electrostatic repulsion between negative charge anions of TC and negative charge catalysts. These findings show that pH plays a significant role in regulating the overall processes and the generation of ROS in the photo-catalytic degradation of metal, in agreement with studies done on metal-supported semiconductors. The TC degradation was investigated at different catalyst dosages (5–40 mg) of ZnO, AgNPs, and AgNPs@ZnO constant concentration of the TC solution (50 mg L^−1^) and a constant pH (4), and an illumination period (120 min).

The experiments with using catalysts produced the results shown in Fig. 12(b). With the ZnO, the rate of degradation was proportional to the quantity of the catalyst, and the higher the quantity of the catalyst used, the higher the rate of degradation was, which was 22 percent at 5 mg of catalyst and 83 percent at 40 mg of catalyst. The increase in the number of active sites and the increase in the generation of ROS are what generate the increase in the degradation efficiency of the catalyst with the increase in its quantity. However, the catalytic value did not rise remarkably with the concentration, and the degradation rate reached the maximum of 40 mg, 77% at 1 mg, 20, and 30 mg, respectively, and was higher than that of the respective AgNPs@ZnO. AgNPs of 5 and 10 mg gave similar results with performance of 9 and 40 percent, respectively, and 20 mg gave 18%, lower than ZnO and AgNPs@ZnO. This weak activity is likely to be attributed to reduced specific surface area and the insufficient capacity of the ROS generation of the AgNPs in the visible light, which will affect the process of degradation. Generally, AgNPs@ZnO NC was highest with a photo-catalytic efficiency of 99% when compared to 28% at 5 mg. This higher performance is attributed to enhanced charge separation as a result of the plasmonic effect of Ag and the high photocatalytic activity of ZnO. All these effects contribute to the formation of ROS and the contact of pollutants, and the increased degradation performance.

However, further doses of catalysts beyond 40 mg might not give a proportional increase. The increase in catalyst loading may lead to the agglomeration of the entire catalyst particles, leading to shielding effects and light scattering, which reduces the effective surface area and the photon absorption. Based on that, the amount of 40 mg of AgNPs@ZnO as a catalyst was chosen as the most appropriate one in the experiment of the current work. These findings are in line with other past studies, which have established that NC-based catalysts are more active than single catalysts, attributed to improved charge carrier dynamics, dominant light harvesting, and a viable number of active sites.

#### 3.8.3. Thermodynamic parameters

The energy impact and possibility of the photocatalytic degradation process of TC on AgNPs@ZnO was studied by computing the thermodynamic parameters based on the Eyring–Polanyi equation derived as a result of the transition state theory:6
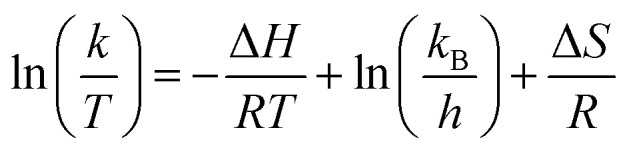
where *k* is the rate constant, *T* is the temperature in Kelvin, *R* is the gas constant (8.314 J mol^−1^ K), *k*_B_ denotes Boltzmann's constant (1.38 x 10–23 J K^−1^), and *h* represents Planck's constant (6.626 × 10–34 J s). This plot yielded a straight line, and the activation enthalpy (Δ*H*) as well as entropy (Δ*S*) have been calculated from its slope and intercept. The Gibbs free energy (Δ*G*) was then calculated using:

7 Δ*G* = Δ*H* − *T*Δ*S*

Values of thermodynamic parameters are displayed in Table 2.

**Table 2:** Values of thermodynamic parameters

Temperature (K)	Rate constant (k min^−1^)	Δ*H* (kJ mol^−1^)	Δ*S* (J mol^−1^ K^−1^)	Δ*G* (kJ mol^−1^)
318	0.06822	2.32	−78.4	27.28
338	0.06822	—	—	28.79
360	0.05915	—	—	30.01
368	0.05307	—	—	30.65

The values of DG at any temperature were positive and therefore indicated that the reaction of degradation of the TC in the dark was not spontaneous. However, with the presence of solar energy, there are sufficient photons to excite electrons, and the reaction ensuing takes place. The small value of DH (2.32 kJ mol^−1^) indicates that the photo-catalytic process is weakly endothermic (This is in favour of the reaction being spurred by the solar or thermal energy). The negative sign of the DS (−78.4 J mol^−1^ K^−1^) was a transition of a rather disordered solution of reactants to a more structured activated complex on the catalyst surface, which is commonly seen in surface-mediated photo-catalytic reactions.

#### 3.8.4. Photo-catalytic degradation mechanism analysis

**Fig. 13:**
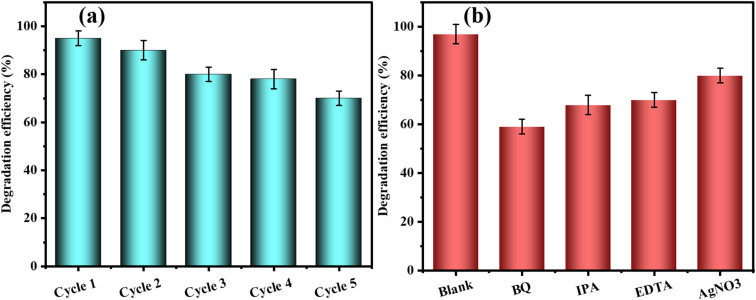
(a) The recyclability of AgNPs@ZnO catalyst for tetracycline degradation under visible light for 5 cycles (b) Effect of different scavengers on the photo-catalytic degradation efficiency of tetracycline by AgNPs@ZnO.

The practicability and stability of the AgNPs@ZnO photo catalyst in the long run were determined by recycling the catalyst five times to degrade the TC under the irradiation of the visible light. As shown in Fig. 13(a), the photo catalyst exhibited a large degradation rate, which was reduced by a small value with each cycle. The first cycle resulted in almost total (100 percent) degradation of TC. However, it was found that there was a progressive performance decline with repetition; the efficiencies had a decline to about 92, 89, 83, and 77 in the second, third, fourth, and fifth cycles, respectively. This minor reduction could be related to partial inactivation of the active sites triggered by the contamination of the surfaces or deposition of the intermediate species or gentle agglomeration of the NPs. Nevertheless, the catalyst did not lose a significant portion of its photo-catalytic capability, and it displayed high stability and reusability, which are also major factors in its practical application in wastewater discharge.

To have a better understanding of the photo-catalytic mechanism and to determine the primary ROS that causes TC degradation, a scavenger study was also performed, which is provided in Fig. 13(b). There was a series of quenching reagents, including (benzoquinone) BQ for (˙O_2_^−^), (isopropyl alcohol) IPA for (˙OH), and EDTA (h^+^). The rate of degradation diminished drastically with BQ, which showed that the superoxide radicals are the active species in the degradation of tetracycline. The presence of IPA and EDTA led to a lesser reduction in the activity, implying the auxiliary role of the ˙OH and h^+^ in the degradation system.

Also, the presence of AgNO_3_ led to a significantly reduced efficiency due to the potential interference of the redox process or electron trapping competition. The findings demonstrate that the photo-catalytic degradation of TC by AgNPsZnO ultimately adheres to a multi-radical mechanism, and ˙O_2_^−^ radicals control the whole reaction.

**Fig. 14:**
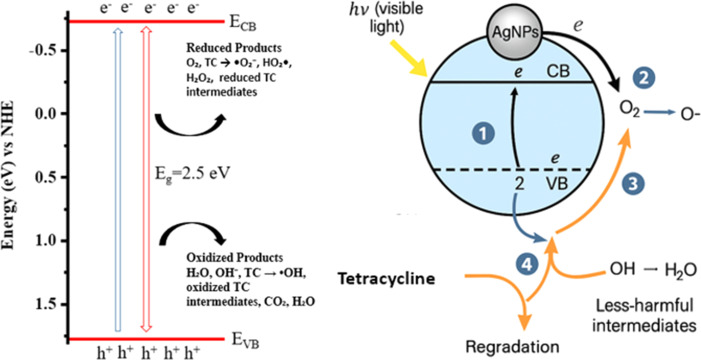
The energy band diagram and photo-catalytic mechanism of AgNPs@ZnO in the presence of visible light associated with the generation of photoinduced e^−^/h^+^ pairs, charge transfer pathways, as well as redox processes during degradation of tetracycline (2.5 eV).

Fig. 14 Flow chart of the proposed mechanism of the photo-catalytic decomposition of tetracycline over AgNPs at ZnO in the face of visible light. Upon irradiation by photons with an energy that matches or exceeds the band gap, electrons are excited out of ZnO in the valence band (VB) to the conduction band (CB), leaving holes in the VB as a pair. The pre-existing Ag nanoparticles act as electron acceptors and facilitate the transfer of photo-generated electrons of the CB of ZnO to the AgNPs surface. This effective separation of charge carriers reduces their recombination, increasing the generation of reactive species. The electron transferred is subsequently applied to decrease the adsorbed oxygen molecules to produce superoxide radical (˙O_2_^−^), which is reactive to organic pollutants. Conversely, the VB loses electrons to O_2_ and water molecules present on the surface of the ZnO to produce O_2_^−^ and hydroxyl molecules, respectively 78); O_2_^−^ loses another electron, and the remaining hydroxyl molecules lose two electrons, which is significant to produce the weak acid form of hydroxyl radicals (OH˙). These ROS then work together to destroy the TC species through gradual degradation through more harmless steps to the ultimate mineralization to CO_2_ and H_2_O. The overall photo-catalytic activity of the AgNPs is enhanced by the presence of AgNPs, which enhances the separation process of photo-generated charges and interfacial electron transfer.

Postulated photocatalytic process in TC Degradation: a step-by-step degradation mechanism of TC on AgNPs@ZnO is as follows:

##### 3.8.4.1. Photon absorption and charge generation

The as-prepared AgNPs@ZnO photo catalyst is stimulated under solar light of energy ≥ band gap, VB–CB excitation takes place with the generation of holes:

8 AgNPs@ZnO + *hv* → AgNPs@ZnO[e_CB_^−^ + h_VB_^+^]

##### 3.8.4.2. Production of superoxide radical

The CB electrons excited by light reduce O_2_ to produce superoxide anion radicals:9
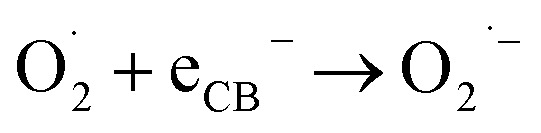


##### 3.8.4.3. Production of the hydroxyl radical

VB h^+^ can neutralize water or hydroxide ions to form HO radicals:

10 H_2_O + h_VB_^+^ → OH˙+H^+^

##### 3.8.4.4. The hydroperoxyl and hydrogen peroxide production

Protons reduce ˙O_2_^−^ to form ˙H_2_O radical cells that further reacts to produce H_2_O_2_ and O_2_:11
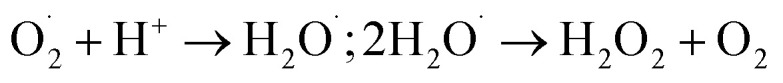


##### 3.8.4.5. Additional generation of hydroxyl radicals

H_2_O_2_ decomposes to form additional ˙OH:

12 H_2_O_2_ + e^−^ → OH˙ + OH^−^

##### 3.8.4.6. Direct reduction and oxidation of TC

Electrons in the CB can reduce TC, electrons can undergo oxidation of TC molecules, and holes in the VB can oxidize TC to form reduced and oxidized intermediates:

13 TC + h^+^ → degraded intermediate

### 3.9 Electronegativity model-based band edge potential estimation

The band edge potentials were determined by way of Butler–Ginley models.

14 *E*_CB_ = *X* − *E*_C_ − 0.5 *E*_g_

15 *E*_VB_ = *E*_CB_ + *E*_g_

Here, *E*_C_ (free electron energy) ¼ 4.5 eV, and *E*_g_ (band gap) was derived from the Tauc plot, *X* (Mulliken absolute electronegativity) was assumed to be 4.439 eV for Ag–ZnO. Using these values, *E*_CB_ and *E*_VB_ were determined as:16
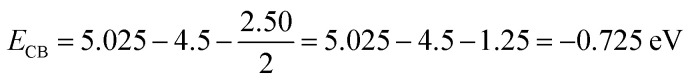

17 *E*_VB_ = −0.725 + 2.50 = +1.775 eV

These band edge positions indicate that the photo-excited electrons at the CB attain sufficient potential energy to reduce O_2_ to ˙O_2_^−^ (the redox potential for O_2_/˙O_2_^−^ is −0.33 eV) and are consistent with our scavenger test results.

An optical band gap of 2.50 eV was obtained from the degradation of TC, a broad-spectrum antibiotic, under visible light irradiation photocatalyst model (AgNPs@ZnONPs hetero-structure used), and the various values were deduced using the Mulliken electronegativity model. The conduction band (CB) and valence band (VB) edge potentials of −0.73 eV (*vs.* NHE), +1.78 eV *vs.* NHE were calculated using the average electronegativity of composites (*X* = 5.025 eV).18
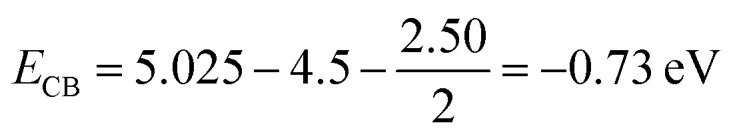

19 *E*_VB_ = −0.73 + 2.5 = +1.78 eV

## 4. Conclusion

A new bimetallic AgNPs@ZnO NC was synthesized and characterized, and it was demonstrated to act as a dual functional NC for photo-catalytic degradation of TC and low temperature HER under solar light. Ag NPs loaded ZnO showed a significant increase in their optical absorbance, decreasing the band gap energy, and enhancing the separation of charge carriers owing to the LSPR effect. The foregoing changes contributed to efficient visible light utilization and resulted in excellent TC-degradation efficiency and highly efficient hydrogen evolution. Optimum conditions involving 20 mg of catalyst usage and neutral pH were determined through parametric studies for maximum degradation. Kinetic study indicated that degradation followed a pseudo-first-order model, and thermodynamic analysis confirmed the process to be spontaneous and endothermic. Radical scavenger tests indicated that superoxide (˙O_2_^−^) and hydroxyl (˙OH) radicals were the predominant active substances for the photo-catalytic degradation. Reusability and photostability over several cycles of the NC were also found to be good, highlighting its strong stability and practical application. All in all, the excellent photo-catalytic performance and H_2_ generation activity make AgNPs@ZnO a cheap, green, and dual-functional photo catalyst for environmental purification and clean energy production. To further assess photon efficiency and mineralization efficiency, the AQY and TOC analysis will be looked into in future work for the purpose of making them comparable with those reported for Ag/ZnO photo-catalytic systems. Scale-up of the process, long-term operation, and application to actual wastewater treatment plants are subjects of further study.

## Author contributions

Sayyar Ali Shah: methodology, investigation, formal analysis, writing – original draft. Hanbing Song: writing – review & editing, formal analysis. Abida Batool: writing – review & editing, formal analysis. Muhammad Saad Riaz: writing – review & editing, formal analysis. Azhar Abbas: conceptualization, supervision, project administration, writing – review & editing, formal analysis. Shoaib Akhtar: writing – review & editing, formal analysis. Umar Nishan: writing – review & editing, formal analysis. Ibrahim A. Shaaban: writing – review & editing, formal analysis. Shah Faisal Mohammad: writing – review & editing, formal analysis.

## Conflicts of interest

The authors declare that they have no known competing financial interests or personal relationships that could have appeared to influence the work reported in this paper.

## Supplementary Material

RA-016-D5RA09874B-s001

## Data Availability

The data supporting this article can be accessed through the link 10.6084/m9.figshare.31869334. Supplementary information (SI): UV-Vis, XRD, FTIR, and TGA. See DOI: https://doi.org/10.1039/d5ra09874b.
