# Supplementary material for: Dual-function silver nanoparticles catalyst supported on ZnO nanoparticles from plant extract: high-TOF hydrogen generation and fast photocatalytic tetracycline removal
Source: RSC Adv. 2026 Apr 1;16(20):17690–711. doi: 10.1039/d5ra09874b (PMC13040451; doi:10.1039/d5ra09874b)
Supplement: RA-016-D5RA09874B-s001 [file RA-016-D5RA09874B-s001.pdf]

Supplementary Information (SI) for RSC Advances.
This journal is © The Royal Society of Chemistry 2026

Dual-Functional Silver Nanoparticles Supported on ZnO Nanoparticles Catalyst from Plant Extract: High-TOF Hydrogen Generation and Fast Photocatalytic Tetracycline Removal

Sayyar Ali Shah[a], Shah Faisal Mohammad[a], Abida Batool[b], Muhammad Saad Riaz[c], Azhar Abbas[c,d,], Shoaib Akhtar[c,*], Ibrahim A. Shaaban[e], Umar Nishan[f], Hanbing Song[a],*

[a]*School of Medical Science, Shandong Xiehe University, Jinan, Shandong Province, P.R.China*

[b]*Department of Chemistry, Superior University Lahore, Lahore 54000, Pakistan*

[c]*Institute of Chemistry, University of Sargodha, Sargodha 40100, Pakistan*

[d]*Government Ambala Muslim Graduate College, Sargodha 40100, Pakistan*

[e]*Department of Chemistry, Faculty of Science, Research Center for Advanced Materials Science (RCAMS), King Khalid University, P.O. Box 960, Abha, 61421, Saudi Arabia.*

[f]*Department of Chemistry, Kohat University of Science and Technology, Kohat 26000 KP Pakistan*

** Corresponding authors:*

Dr Azhar Abbas: Govt. Ambala Muslim Graduate College, Sargodha, 40100, Pakistan;

https://orcid.org/0000-0002-5741-7136*: Email:* azhar.ramzan@uos.edu.pk

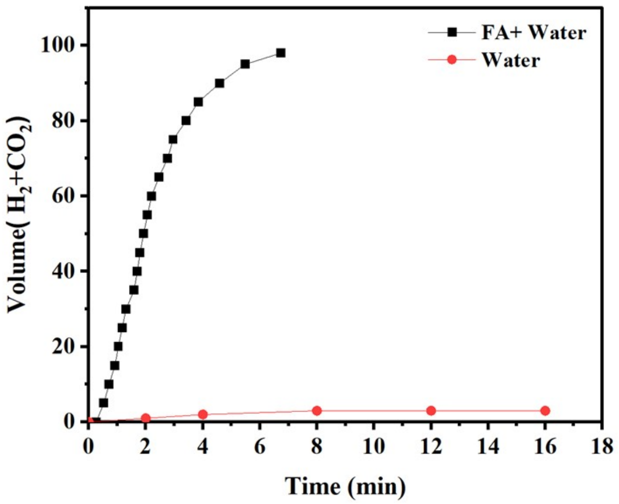

Fig. S1. The plot of Volume of gases evolved (CO_2 + H_2) vs time

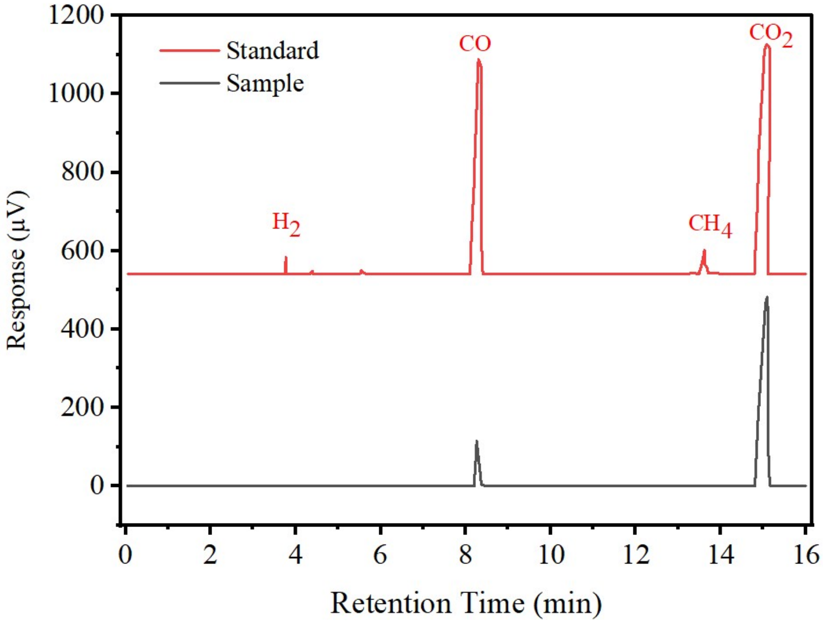

Fig. S2. GCTD analysis of evolved gases

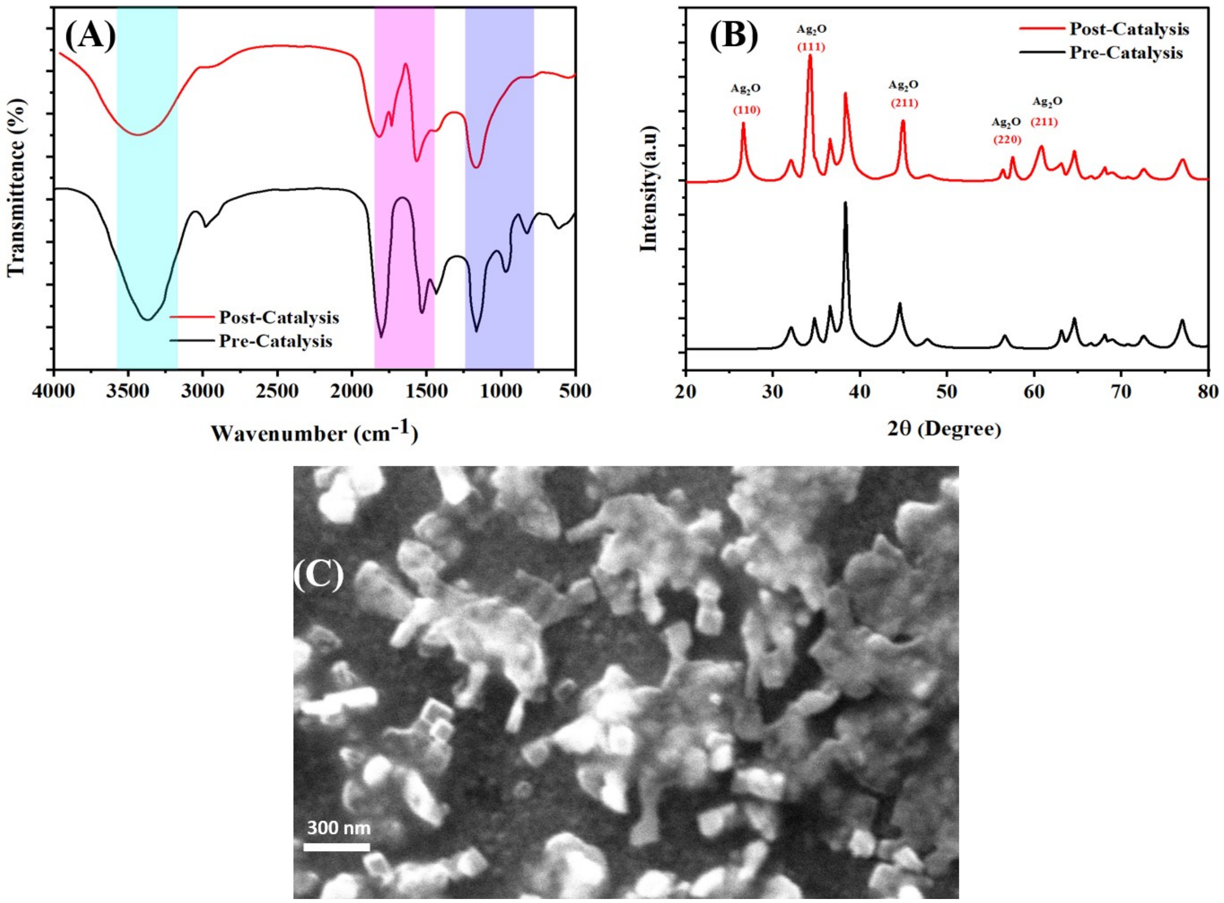

Fig. S3. Post HER (A) FTIR, (B) XRD, and (C) SEM image of the catalyst

The data supporting this article (UV-Vis, XRD, FTIR, and TGA) can be accessed through the link 10.6084/m9.figshare.31869334.
